# A Super‐Resolution Approach for Astrocyte‐Specific Molecular Imaging Reveals the Nanoscale Distribution of Monoacylglycerol Lipase, the Metabolic Node Between Endocannabinoid and Prostaglandin Signaling

**DOI:** 10.1002/glia.70186

**Published:** 2026-07-03

**Authors:** Miklós Zöldi, István Katona

**Affiliations:** ^1^ Department of Psychological and Brain Sciences Indiana University Bloomington Indiana United States; ^2^ Laboratory of Molecular Neurobiology HUN‐REN Institute of Experimental Medicine Budapest Hungary; ^3^ School of Ph.D. Studies Semmelweis University Budapest Hungary

**Keywords:** astrocyte, endocannabinoid, MAGL, mgll, prostaglandin, STORM, super‐resolution imaging

## Abstract

Astrocytes play essential roles in brain function and disorders. Yet, compared to neurons, our knowledge of the physiological and pathological signaling mechanisms in astrocytes remains limited. As a major challenge, the ultrathin (~10–100 nm) processes of astrocytes render high‐throughput quantitative molecular imaging within well‐defined cellular contexts very difficult. Here, we introduce a single‐molecule localization microscopy‐based methodology that achieves unprecedented resolution of the intricate astrocytic arbor in intact brain circuits. Postnatal tagging of the plasma membrane by electroporation in mice resulted in selective and sparse labeling of hippocampal astrocytes and enabled the complete visualization of individual astrocytes with nanoscale precision by using STochastic Optical Reconstruction Microscopy (STORM). We also developed high‐yield and easy‐to‐implement approaches to segment, measure, analyze, and visualize nanoscale molecular information within astrocytic compartments. As a proof‐of‐concept, we could readily differentiate between synaptic and astrocytic proteins by using dual‐color STORM super‐resolution imaging. Moreover, we identified cell‐type‐specific differences in the distribution of monoacylglycerol lipase (MAGL), an enzyme regulating synaptic plasticity in neurons and coupling endocannabinoid signaling to prostaglandin signaling in astrocytes. Our findings demonstrate the feasibility of nanoscale molecular measurements within ultrathin astrocytic processes. Moreover, the results provide insights into the synapse‐independent nanoscale arrangement of the astrocytic MAGL pool that controls neuroinflammatory processes.

## Graphical abstract

- Dual‐color STORM resolves the precise architecture and protein distributions within hippocampal astrocytes at the nanoscale.
- Identification of a sparse but specific MAGL pool in CA1 astrocytic processes.
- Astrocytic MAGL is not synapse‐associated, suggesting a synapse‐independent role.

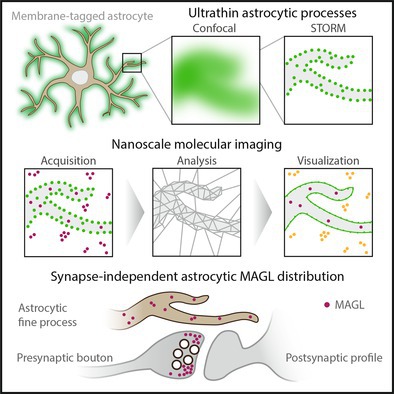

## 1 Introduction

Although astrocytes are nearly as numerous as neurons (Khakh 2025), the full spectrum of their vital roles in most, if not all, physiological processes in the brain has only recently gained widespread recognition. Emerging evidence also illuminates their previously underappreciated molecular diversity and morphological complexity that are likely to serve versatile astrocytic functions ranging from the regulation of synaptic communication to network oscillations (Dallérac et al. 2018; Khakh and Deneen 2019; Endo et al. 2022; Oliveira and Araque 2022; Khakh 2025). Moreover, astrocyte‐specific molecular and anatomical changes are associated with abnormal astrocytic activity in most brain disorders, especially in the context of neuroinflammatory processes (Linnerbauer et al. 2020; Diaz‐Castro et al. 2021; Hasel et al. 2021; Brandebura et al. 2023; Lee et al. 2023). However, quantitative molecular and morphological measurements in astrocytes represent a major methodological challenge (Yu et al. 2020; Baldwin et al. 2024). For example, protoplasmic astrocytes exhibit a highly compartmentalized and convoluted morphology (Arizono and Nägerl 2022; Baldwin et al. 2024; Chiappini et al. 2026). While complex processes such as branchlets, leaflets and perisynaptic astrocytic processes represent ~90% of their surface area, the small diameter (< 100 nm) of most of these processes is below the resolution limit of confocal microscopy (Ventura and Harris 1999; Reeves et al. 2011; Baldwin et al. 2024). Ensemble super‐resolution techniques offer substantially improved insights into astrocyte morphology (Arizono et al. 2020; Valli et al. 2021; Salmon et al. 2023; Baldwin et al. 2024) but have not been optimized for high‐yield and quantitative nanoscale molecular imaging in ultrathin astrocytic processes. Volume electron microscopy approaches have recently been successfully used to provide unprecedented details of the nanoarchitecture of astrocytes (Aten et al. 2022; Salmon et al. 2023; Benoit et al. 2025). However, electron microscopy requires strong tissue fixation thereby limiting antibody penetration and reducing molecular labeling density. This is important, because the expression levels of several functionally relevant astrocytic transcripts are low compared to neurons, and proteomics data often predict even lower abundance for these targets at the protein level (Cahoy et al. 2008; Yu et al. 2020).

Single‐molecule localization microscopy (SMLM), a group of super‐resolution imaging modalities that rely on the nanoscale localization of individual fluorophores (Lelek et al. 2021), represents a breakthrough for molecular and cellular neuroscience (Dani et al. 2010; MacGillavry et al. 2013; Nair et al. 2013; Specht et al. 2013; Baddeley and Bewersdorf 2017; Biederer et al. 2017; Sauer and Heilemann 2017). As a major advantage, due to single‐molecule detection sensitivity, SMLM approaches capture low copy number proteins and probe molecular heterogeneities. Moreover, the sample preparation steps are compatible with traditional light microscopy‐based techniques ensuring efficient tissue penetration of antibodies and simultaneous imaging of multiple target proteins. On the other hand, in contrast to electron microscopy, the cellular membranes that are required for the unequivocal assignment of nanoscale molecular data to specific biological structures are not visualized in SMLM approaches. We have shown that correlated confocal microscopy and STORM imaging make quantitative molecular imaging in a microscale‐sized subcellular context feasible (Dudok et al. 2015; Barna et al. 2016; Zöldi and Katona 2023; Barti et al. 2024). However, the proper delineation of the sub‐microscale astrocytic processes would require near‐molecular resolution that is beyond the diffraction limit of confocal microscopy (Salmon et al. 2023; Soto and Khakh 2023), imposing a challenge for correlated visualization of astrocytic membranes at the sub‐diffraction level together with target protein imaging at the nanoscale level.

Besides their fundamental roles in regulating neuronal processes such as synaptic transmission, astrocytes are also central players in neuroinflammatory mechanisms driving neurodegeneration (Linnerbauer et al. 2020; Lee et al. 2023). The monoacylglycerol lipase (MAGL) enzyme has recently emerged as a central molecular player coupling synaptic and inflammatory processes (Chen 2023; Malhotra et al. 2025). Inhibition of MAGL activity promotes 2‐arachidonoylglycerol (2‐AG) endocannabinoid‐mediated retrograde synaptic signaling, thereby attenuating excitatory neurotransmission and preventing hyperexcitability (von Rüden et al. 2015; Terrone et al. 2018; Farrell et al. 2021). Moreover, MAGL inhibition also impedes hypoxia and subsequent neurodegeneration by restricting PGE_2_ prostaglandin production (Nomura et al. 2011; Pan et al. 2011; Farrell et al. 2021). Accordingly, MAGL inhibitors have shown promising effects in preclinical models of stroke, traumatic brain injury, bacterial endotoxin‐mediated brain inflammation, autoimmune encephalomyelitis, Alzheimer's disease, Huntington's disease, and Parkinson's disease (Nomura et al. 2011; Chen et al. 2012; Piro et al. 2012; Alhouayek et al. 2014; Grabner et al. 2016; Choi et al. 2018; Ruiz‐Calvo et al. 2019; Hu et al. 2022; Guadalupi et al. 2024). Interestingly, while the MAGL protein is abundant in presynaptic axon terminals (Gulyas et al. 2004; Ludányi et al. 2011), MAGL activity in astrocytes also plays important roles in both synaptic plasticity (Tanimura et al. 2012; Chen et al. 2016; Liu et al. 2016; Zhu, Zhang, Gao, et al. 2023) and neuroinflammatory processes (Viader et al. 2015; Zhu, Zhang, Hashem, et al. 2023; Sun et al. 2025). The broad spatial and temporal spectrum of MAGL function that extends from restricting synapse‐specific plasticity processes to circuit‐level neurovascular coupling (Farrell et al. 2021; Dudok et al. 2024) hence raises the intriguing question of how cell‐type‐ and subcellular compartment‐specific MAGL pools are organized to subserve these complex physiological processes.

To address the methodological challenge of quantitative nanoscale molecular imaging in ultrathin astrocytic processes, we developed an efficient approach that achieves an unprecedented diffraction‐unlimited resolution of astrocyte morphology and enables nanoscale molecular measurements even in astrocytic leaflets and perisynaptic processes. We also introduce a workflow for control experiments, data analysis, and data visualization. Finally, we present the distinct distribution of the neuronal and astrocytic MAGL enzyme pools, providing insights into the structural basis of the synapse‐specific and synapse‐independent physiological and pathological functions of MAGL.

## 2 Materials and Methods

### 2.1 Animals

Animal experiments were approved by the Hungarian Committee of the Scientific Ethics of Animal Research (license number: PE/EA/201‐7/2020) and were carried out according to the Hungarian Act of Animal Care and Experimentation (1998, XXVIII, Section 243/1998, renewed in 40/2013), which is in accordance with the European Communities Council Directive of November 24, 1986 (86‐609‐EEC; Section 243/1998). Mice were kept under approved, specific‐pathogen‐free laboratory conditions, and all efforts were made to minimize pain and to reduce the number of animals used. To develop the methodology for the PALE approach and astrocyte‐specific STORM imaging in the CA1 subregion of the hippocampus, we used male and female CD‐1 mice (ranging in age from 29 to 155 days; Charles River Laboratories Hungary). To study the nanoscale distribution of MAGL and to verify the specificity of the MAGL antibody, we used male *Mgll* ^+/+^ and *Mgll* ^−/−^ littermates (age: 100–166 days, kindly provided by Prof. Kenji Sakimura, Niigata University) that were kept on a C57BL/6N background. Mice were bred and genotyped as previously described (Uchigashima et al. 2011). RNAscope experiments utilized 60–66‐day‐old mice from a distinct MAGL line (Schlosburg et al. 2010), which was generously provided by Prof. Benjamin F. Cravatt (The Scripps Research Institute) and Prof. Anna Kalinovsky (Indiana University). These specific procedures were approved by the Institutional Animal Care and Use Committee (IACUC) of Indiana University (protocol number: 21‐015) and conform to the National Institutes of Health Guidelines on the Care and Use of Animals.

### 2.2 Postnatal Astrocyte Labeling by Electroporation (PALE)

Newborn and postnatal day 1 (P0‐P1) pups were sedated by hypothermia. Glass capillaries were pulled by a micropipette puller (Sutter Instrument, Novato, CA, USA) and were filled with plasmid DNA solution (diluted in endotoxin‐free water to 1 μg/mL concentration). Injection was delivered into one of the lateral ventricles of the pups. Using mouth aspirator tubes (Sigma‐Aldrich, A5177), 1–2 μL of plasmid solution was administered into the ventricles. The non‐toxic Fast Green dye (1:10,000; Roth) was added to the solution to visualize the crescent‐shaped ventricle and to determine the success of the injections. Electroporation was performed with tweezer electrodes using an SP‐3c electroporator (Supertech, adjusted to 5 pulses of 100 V for 50 ms at 950 ms intervals). The negative terminal of the electrode was placed above the site of injection, while the positive terminal was placed to the opposite side of the head, below the pup's chin. The two terminals of the electrodes were slightly rotated during electroporation to ensure a homogeneous labeling of the entire CA1 region of the hippocampus. For targeting cortical astrocytes, the electrode polarity was reversed. After electroporation, the pups were placed onto a thermal heating pad until their skin color recovered and then were returned to their dams. When performing PALE on two consecutive days, the same procedure was repeated on P0 and P1 on the same pups. No pups died because of the electroporation procedure. Electroporation on P2 did not achieve the “useful density” of PALE‐labeled astrocytes. We defined “useful density” as at least 10 astrocytes per 20 μm‐thick section. The extent of labeled astrocytes along the transverse axis of the hippocampus (i.e., along the CA1–CA3 trajectory) spanned a mean of 1962 μm (SD = 380 μm). The range of successfully labeled astrocytes along the rostro‐caudal axis in adjacent sections was approximately 1000–1500 μm. Expression of the injected construct was observed as early as P5, and expression levels remained high up to 227 days of age, the oldest time point investigated in this study.

### 2.3 DNA Constructs

The following constructs were used: pAAV‐GFAP104‐ChR2‐mCherry plasmid (Addgene #58892); CAG‐ChR2‐GFP (Addgene #26929); Lck‐GFP (Addgene #61099); and GFAP‐EGFP (Addgene #50473). The constructs visualized a similar number of astrocytes with comparable morphological features. Because radial glia primarily differentiate into astrocytes around the time of birth, the use of astrocyte‐specific promoters is not strictly required for postnatal electroporation to achieve cell type‐specific targeting. ChR2 was not used for optogenetic activation of astrocytes; it only served (similarly to the Lck tag) to ensure the integration of the fluorescent protein‐containing construct into the plasma membrane. We preferred mCherry over GFP to visualize astrocytes, as mCherry could be more easily bleached. This allowed us to reuse the same spectral channel for STORM imaging with the STORM‐compatible CF568 dye.

### 2.4 Preparation of Hippocampal Sections

Two approaches were used in this study. The two tissue preparation methods did not result in notable differences in the immunolabeling pattern. In the first approach, mice were deeply anesthetized with an intraperitoneal injection of Avertin (1.25% v/v, Sigma‐Aldrich) and were perfused transcardially with 0.9% saline for 2 min and then with 4% paraformaldehyde (PFA, TAAB, P001) dissolved in 0.1 M phosphate buffer (PB, pH = 7.4 containing Na_2_HPO_4_, Sigma‐Aldrich, 304,305 and NaH_2_PO_4_, Sigma‐Aldrich, 71,500) for 20 min using a peristaltic pump at 5 mL/min speed. Brains were removed from the skull and postfixed overnight in 4% PFA. Hippocampal coronal sections were cut on a Leica VT‐1200S vibratome at thicknesses of 20 μm for STORM imaging and 50 μm for confocal microscopy.

In the second approach, acute hippocampal slices were prepared in the same manner as the traditional approach in ex vivo electrophysiological experiments. Briefly, mice were deeply anesthetized by isoflurane. The brains were quickly removed from the skull and transferred to ice‐cold sucrose containing artificial cerebrospinal fluid (ACSF, containing in mM: 75 NaCl, 75 sucrose, 2.5 KCl, 25 glucose, 1.25 NaH_2_PO_4_, 4 MgCl_2_, 0.5 CaCl_2_, 24 NaHCO_3_, Sigma‐Aldrich, St. Louis, MO, USA) equilibrated with 95% O_2_ and 5% CO_2_. The 300‐μm‐thick coronal hippocampal acute slices were cut using a Leica VT‐1200S vibratome. Slices were pre‐incubated at 34°C in sucrose containing ACSF for 1 h. Afterwards, the slices were placed into 4% PFA for overnight immersion fixation at 4°C, and the next day the fixative was changed to 0.1 M PB. To determine the success of the electroporation, slices were mounted onto a microscopic glass slide, covered by a coverslip, and placed under an upright microscope (Nikon Eclipse 80i, equipped with a DS‐Fi1 CCD camera). Slices containing a suitable number of astrocytes were re‐embedded in 2% agarose and cut on a vibratome to 20‐μm‐thick sections. We found the acute slice method better than the perfusion‐based approach for selecting the most suitable sections. In addition, fixation quality during perfusion introduces additional variability in immunolabeling efficiency. Therefore, the acute slice approach was used in case of comparison of immunolabeling obtained from *Mgll* ^+/+^ and *Mgll* ^−/−^ littermate mouse hippocampi.

### 2.5 Immunolabeling

Immunolabeling of hippocampal sections was carried out in a free‐floating manner in 24‐well tissue culture plates (Greiner Bio‐One CELLSTAR, 662102), gently rotated on orbital shakers (Biosan OS‐10). Sections were blocked and permeabilized with 5% normal donkey serum (NDS) and 0.3% Triton in Tris‐buffered saline (TBS, pH = 7.4, 0.05 M, containing Trizma hydrochloride, Trizma base and NaCl) for 45 min. Sections were incubated overnight at 4°C in primary antibody solution diluted in TBS. After washing the sections 3 times 10 min in TBS, incubation in the respective secondary antibody solution was carried out for 4 h. For primary and secondary antibodies and their concentrations, see Table 1. Sections were washed again 3 times for 10 min in TBS, then in 0.1 M PB. For confocal imaging, sections were mounted on glass microscope slides with Vectashield mounting medium (Vector Laboratories, H‐1200‐10), covered with coverslip and sealed with nail polish. For super‐resolution imaging, the sections were postfixed with 4% PFA for 10 min, washed again 3 times for 10 min in 0.1 M PB and mounted onto coverslips.

**TABLE 1:** List of primary and secondary antibodies used in the study.

Antibody	Source	Identifier	Dilution
Guinea pig anti‐MAGL	Frontier Institute	Cat# MGL‐GP‐Af200; RRID: AB_2716807	1:200
Rabbit anti‐RFP	Rockland	Cat# 600–401‐379: RRID: AB_2209751	1:2000
Mouse anti‐RFP	Rockland	Cat# 200–301‐379; RRID: AB_2611063	1:2000
Goat anti‐GFP	Abcam	Cat# ab5450; RRID: AB_304897	1:2000
Guinea pig anti‐GLT‐1	Millipore	Cat# AB1783; RRID: AB_90949	1:1000
Rabbit anti‐Glutamine Synthetase	Abcam	Cat# ab49873; RRID: AB_880241	1:10000
Rabbit anti‐MAP2	Millipore	Cat# AB5622; RRID: AB_91939	1:1000
Mouse anti‐Bassoon	Abcam	Cat# ab82958; RRID: AB_1860018	1:3000
Mouse anti‐GFAP	Millipore	Cat# MAB3402; RRID: AB_94844	1:1000
Rabbit anti‐GFAP	Millipore	Cat# AB5804; RRID: AB_2109645	1:1000
Mouse anti‐NeuN	Millipore	Cat# MAB377; RRID: AB_2298772	1:500
Rabbit anti‐Iba1	FUJIFILM	Cat# 019–19,741; RRID: AB_839504	1:2000
Alexa 488 donkey anti‐mouse	Jackson	Cat# 715–545‐150; RRID: AB_2340846	1:400
Alexa 488 donkey anti‐rabbit	Jackson	Cat# 711–545‐152; RRID: AB_2313584	1:400
Alexa 594 donkey anti‐mouse	Jackson	Cat# 715–585‐150; RRID: AB_2340854	1:400
Alexa 594 donkey anti‐rabbit	Jackson	Cat# 711–585‐152: RRID: AB_2340621	1:400
Alexa 647 donkey anti‐mouse	Jackson	Cat# 715–605‐150; RRID: AB_2340862	1:400
Alexa 647 donkey anti‐rabbit	Jackson	Cat# 711–605‐152; RRID: AB_2492288	1:400
Alexa 647 donkey anti‐goat	Jackson	Cat# 705–605‐147; RRID: AB_2340437	1:400
Alexa 647 donkey anti‐guinea pig	Jackson	Cat# 706–605‐148; RRID: AB_2340476	1:400
CF 568 donkey anti‐mouse	Biotium	Cat# 20105; RRID: AB_10853136	1:1000
CF 568 donkey anti‐rabbit	Biotium	Cat# 20098; RRID: AB_10557118	1:1000

The dual‐color STORM imaging experiment demonstrated that there was no antibody cross‐reactivity for the MAGL antibody in our experimental settings. As a word of caution, we noted that the MAGL antibody lost its efficacy upon storage for 5–6 months and cross‐reaction appeared with other primary antibodies. We observed this phenomenon multiple times and with different storage conditions. Therefore, we only used fresh, few weeks‐old antibody batches in our MAGL‐immunolabeling experiments.

### 2.6 RNAscope In Situ Hybridization

*Mgll* ^+/+^ and *Mgll* ^−/−^ littermate mice were deeply anesthetized by isoflurane and perfused with 4% PFA. The brains were sectioned to 50‐μm‐thick sections and mounted on positively charged microscopic glass slides (Fisher Scientific). The sections were dehydrated in a series of ethanol solutions (50%, 70%, 100%) and kept in 100% ethanol overnight. On the following day, the sections were dried, and a barrier was drawn around them by using a hydrophobic pen (ImmEdge Hydrophobic Barrier, Vector Laboratories). In situ hybridization was performed based on the protocol provided by the manufacturer Advanced Cell Diagnostics (ACD). The RNAscope probes were ordered from ACD (*mgll*, Cat No.: 478831, target region: 2 – 1357; *glul*, Cat No.: 426231‐C3, target region: 103 – 973).

### 2.7 Confocal Imaging

Low‐magnification large images were taken using a 20× objective (CFI Plan Apo VC Air 0.75 NA) on a Nikon A1R confocal laser‐scanning microscope operated by NIS‐Elements AR software 4.50.0.

### 2.8 Single‐Color and Dual‐Color STORM Image Acquisition

For STORM image acquisition and analysis, we followed the workflow described in detail in our earlier protocols (Barna et al. 2016; Zöldi and Katona 2023). Briefly, dried sections mounted on coverslips were covered just prior to imaging in STORM imaging buffer containing thiol‐based switching agent and oxygen‐scavenging system (0.1 M mercaptoethylamine, 5% glucose, 1 mg/mL glucose oxidase, and 1500 U/mL catalase in Dulbecco's phosphate‐buffered saline (DPBS)). For dual‐color STORM imaging, the sections were mounted in Smart buffer (Abbelight). Sections were sealed with nail polish or Twinsil (Picodent), dried for 10 min, and inserted onto the microscopic stage of a Nikon N‐STORM system that was equipped both with a C2 confocal scan head and an Andor iXon Ultra 897 EMCCD camera and was operated by the NIS Elements AR 4.3 software and N‐STORM 3.4 module.

First, to locate the electroporated astrocytes, a low‐magnification large image was taken by using a CFI S Plan Fluor 20× 0.45 NA objective. After switching to a high‐magnification objective (CFI Apo TIRF 100× Oil 1.49 NA), we performed direct STORM (*d*STORM) imaging. Laser powers for the different lines (647 nm, 300 mW, MPB Communications VFL‐P‐300‐647; 405 nm, Melles Griot 56RCS/S2780; 488 nm, Coherent sapphire 488–200 CW; 561 nm, Coherent sapphire 561–100 CW), as well as switching the laser beam between total internal reflection fluorescence (TIRF) and confocal light paths were adjusted by acousto‐optical tunable filters (AOTFs). The focus was stabilized with a perfect focus system (PFS). A cylindrical lens inserted into the light path enabled 3D imaging and the *z*‐coordinates of localization points could be retrieved from calibration curves that were obtained from imaging of fluorescent beads attached to coverslips. Before acquiring the STORM image, fluorophores were pushed to their dark state by illumination with their respective laser lines. Next, we tilted the angle of the laser beam from standard epi‐illumination to near‐TIRF using a TIRF illuminator (equipped also with 2× and 4× beam focusing lens) to reduce the out‐of‐focus fluorescence. For single‐color STORM imaging, the 647 nm laser line was used with a STORM filter cube (DM: 660 nm; EM: 670–760 nm). The reactivation rate of fluorophores was adjusted by increasing the power of the 405 nm activation laser to the desired level without introducing overlapping blinking events. For dual‐color STORM imaging, a QUAD‐band filter cube (Chroma, 97,335) was used in a sequential imaging protocol. First, the target‐of‐interest was imaged by using the 647 nm laser line. Next, we pushed the CF568 fluorophores to their dark state in epi‐illumination by using the 561 nm laser line. Subsequently, the TIRF angle was readjusted, and a STORM image was acquired. We found this sequential imaging strategy superior compared to alternating laser lines in each frame, because the abundant astrocytic CF568 localizations required a few tens of seconds of illumination prior to imaging, a step that would quench most of the AF647 fluorophores.

### 2.9 Correlated Confocal and STORM Imaging

To resolve differences in astrocytic morphological detail across imaging scales, as well as to determine the distribution of astrocytic MAGL localization points in relation to synapses labeled by Bassoon, we first acquired a confocal z‐stack (size: 512 × 512 pixels, pixel size: 80 nm, z‐step size: 150 nm, z‐range: 2 μm) from the very same region of interest that was subsequently imaged by the STORM microscope. Confocal images were deconvolved with Huygens software (SVI) using the CMLE algorithm.

### 2.10 STORM Image Analysis

Peak detection of individual localization events was performed by the N‐STORM module that relies on the 3D‐DAOSTORM algorithm. In case of the sequentially acquired dual‐color images, the drift correction of the last localization detected in the first image (647‐nm channel) was additionally applied for all the localizations of the second image because the software considers the two STORM movies as independent imaging sessions. The level of chromatic aberration between the 647/561 laser lines was assessed in advance on TetraSpeck beads attached onto coverslips and was found to be negligible in *xy* dimensions and ~60 nm in the *z* dimension. Therefore, the *z*‐coordinates of the far‐red channel were shifted by 60 nm. The localization points from both channels were then clipped from the original 800‐nm axial range (±400 nm from the focal plane) to a 600‐nm range (±300 nm from the focal plane), thereby avoiding non‐overlapping regions in the *z* dimension. Note that localizations are detected with a Gaussian probability along the axial dimension, and therefore the relative abundance of localization points may also be slightly shifted axially to each other. Because the 2× TIRF focusing lens introduces inhomogeneity at the edges of the imaging area, we filtered for localizations detected in the center of the camera ROI (corresponding to 10,240 nm^2^, 1/4 of the imaged area).

Custom written Python codes were used in conjunction with various software to analyze the STORM data. To determine the channel crosstalk in STORM images, the astrocytes labeled with a single fluorophore were sequentially imaged using their respective 647‐nm and non‐specific 561‐nm lasers in case of immunolabeling with a far‐red dye‐conjugated secondary antibody and vice versa. Crosstalk was determined as the ratio of the number of non‐specific/specific localization points. Images were also taken from areas where no labeled astrocytes were present. The number of localization points detected here was considered as background and was subtracted from their respective images. When different peak identification thresholds were used to retrieve the number of localization points from the non‐specific illumination, the threshold for the specific illumination was kept constant.

To assess the level of colocalization of dual‐labeled astrocytes, we retrieved the Manders' coefficient values from Voronoi tessellations using Coloc‐Tesseler (Levet et al. 2019). To determine whether there is a systematic bias in the localizations due to chromatic aberration, we shifted the localizations of one channel in *xy* dimensions relative to the other from −60 to 60 nm in 20 nm increments and calculated the Manders' values for all possible offset combinations. We found that the shifts decreased Manders' values, indicating the lack of *xy* chromatic aberration. Following channel shifting, non‐overlapping regions were excluded from both channels to avoid biasing the colocalization analysis by introducing artificial padding. Two‐dimensional Voronoi cells were generated, with the threshold value for object creation set to 1 in both channels. Although the 647‐nm channel contained more localizations because AF647 exhibited superior blinking compared with CF568, Manders' coefficients reflect the degree of co‐occurrence rather than the exact stoichiometric relationship between the two channels. Therefore, these values are relatively insensitive to differences in target density between channels. To assess the co‐occurrence of astrocytic localization points with the protein of interest (GS, Bassoon, GLT‐1, MAP2), we created 3D Voronoi cells in Coloc‐Tesseler. Because many localization points on the edges of relevant structures were discarded with threshold values of 1, we set 0.5 for thresholding both channels. Notably, false colocalizations did not occur with these lower threshold settings. We used AutoHotkey scripts to automate Coloc‐Tesseler for opening the images, adjusting parameters and saving the results.

To filter MAGL localization points belonging to astrocytes, we created 2D Voronoi cells from the astrocytic localization points in SR‐Tesseler (Levet et al. 2015), constructed objects reflecting astrocyte processes with a threshold (density factor) = 1.5 and saved the output image. Then, we evaluated the contours of these objects on the output image using the OpenCV module in Python and determined the scaling factor between the pixelated image and the STORM image to retrieve the coordinates of the astrocytic contours in the STORM coordinate system. The Matplotlib Path module was used to test if a localization point is located inside these polygons and therefore can be assigned as a localization point representing MAGL in astrocytes. To determine if MAGL is enriched inside astrocytes, we rotated the MAGL STORM image relative to the astrocyte STORM image and compared the ratio (mean value from rotations by 90, 180, 270 degrees) of astrocytic/non‐astrocytic points to the ratio obtained from the original image.

To test if astrocytic MAGL blinking events are enriched near synapses marked by Bassoon, we acquired correlated confocal and STORM images. To match the axial range of the STORM localization points, a maximum‐intensity projection (MIP) was generated from the three central optical sections of the deconvolved confocal z‐stacks. The two different imaging modalities were loaded in the VividSTORM software (Barna et al. 2016), the offset between them was determined, and STORM localization points were shifted with this value. Next, Otsu's algorithm was applied to the original Bassoon confocal z‐stack rather than to the MIP. The resulting binary image was converted into a point‐coordinate list by multiplying the voxel indices by the lateral pixel size and axial z‐spacing, thereby obtaining the *x*, *y*, and *z* coordinates of each segmented voxel. MAGL blinking events were identified by DBSCAN (SciPy module, *n* = 5, epsilon = 100 nm). For each astrocytic MAGL blinking event (center of mass), we determined the nearest neighbor distance (NND) of the thresholded Bassoon voxels. Next, while preserving the spatial pattern of individual MAGL blinking events, we randomized their positions 100 times within their respective astrocytic contours. We then calculated the NNDs to Bassoon‐positive voxels for each randomized dataset and compared the cumulative distributions of the original and randomized datasets. NNDs were calculated using the SciPy spatial module in Python.

### 2.11 Statistical Analysis and Figure Preparation

GraphPad Prism 9 software was used for statistical analysis and graph construction. Comparing the distribution of large datasets (nearest neighbor distances of all localization points) using Kolmogorov–Smirnov statistics was done via Python's SciPy package. Figures were produced in Adobe Illustrator. The color and contrast of microscopic images were adjusted in Adobe Photoshop, where images were treated in the same manner for all groups or genotypes. STORM localization points with intensities reflecting localization precision were exported from NIS Elements N‐STORM module after applying density filtering. The Visual Molecular Dynamics (VMD) software was used to visualize molecules as spheres. Single or dual‐color tessellated images were exported from SR‐Tesseler or Coloc‐Tesseler, respectively. AI‐based language models were used exclusively for minor linguistic rephrasing of author‐written text and did not contribute to the scientific content of the manuscript.

## 3 Results

### 3.1 STORM Super‐Resolution Imaging Visualizes the Intricate Architecture of Hippocampal Astrocytes at the Nanoscale Level

Cell‐type‐specific nanoscale molecular imaging requires precise delineation of the cellular contour to enable unequivocal parsing of the super‐resolved molecular data to the target cell type. Plasma membrane‐targeted fluorescent probes provide a complete outline of the individual subcellular profiles (Shigetomi et al. 2013). Because astrocyte labeling at high densities is not optimal for subsequent high‐quality STORM imaging, we turned to the technique called postnatal astrocyte labeling by electroporation (PALE, (Stogsdill et al. 2017)) which was shown to be an effective and easy way to deliver plasmid DNA into a sparse subset of astrocytes in the neocortex.

**FIGURE 1:**
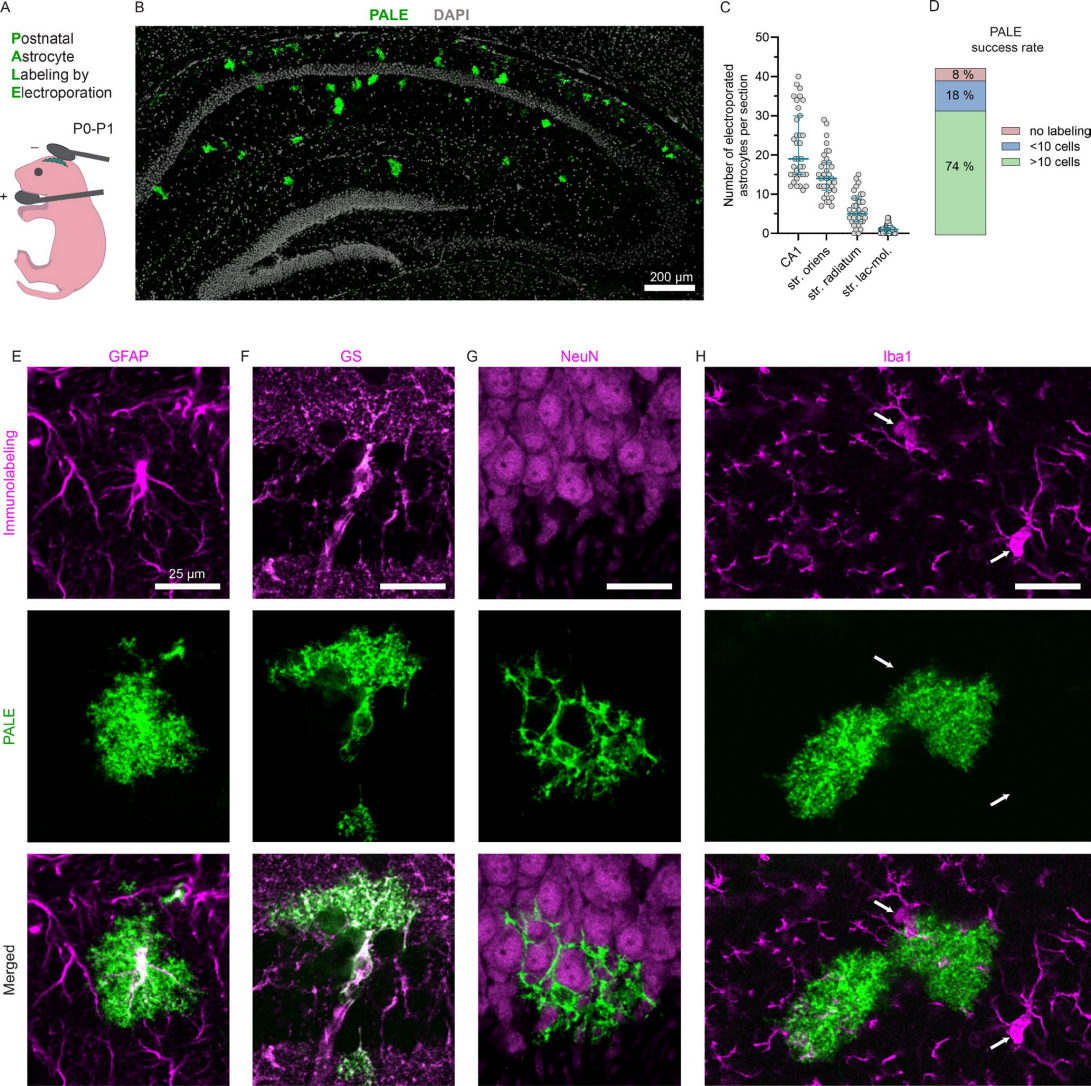
Postnatal astrocyte labeling by electroporation demarcates complex astrocytic arbor topography within dense brain tissue. (A) Schematics of the PALE (Postnatal Astrocyte Labeling by Electroporation) method. (B) Confocal images show that PALE enables sparse and specific labeling of hippocampal astrocytes. (C) Quantification of the labeled cells in the CA1 area in 20–50 μm thick hippocampal sections. Only sections with at least 10 labeled cells were included in this analysis. Blue line shows the median with the interquartile range. (D) Success rate of the hippocampal PALE method from a total of 66 animals from 14 litters (independent electroporations). (E–H) Electroporated astrocytes express the astrocytic markers glial fibrillary acidic protein (GFAP) (E) and glutamine synthetase (GS) (F), but not the neuronal marker NeuN (G) or microglial marker Iba1 (H). For the above markers *n* = 58, 45, 29, and 42 cells were analyzed, respectively. Arrows in (H) highlight microglial cell bodies adjacent to labeled astrocytes.

First, we modified PALE to label hippocampal astrocytes (see Section 2 for details). Briefly, we injected a plasmid encoding a fusion construct of Green Fluorescent Protein and the Channelrhodopsin‐2 protein (ChR2‐GFP) into the lateral ventricles of P0‐P1 pups, carried out electroporation, and investigated the distribution of labeled cells in adult mice. When positioning the positive terminal of the electrode above the skull, astrocytes were labeled in the cortex and along the wall of lateral ventricles (Figure S1A,B), but not in the hippocampus. Interneurons in the olfactory bulb were also visualized (Figure S1C), as described earlier (Boutin et al. 2008; Wang et al. 2013). By reversing the orientation of the two electrodes (Figure 1A, see Section 2), we successfully targeted cells within the hippocampal CA1 subfield. These labeled cells exhibited the characteristic star‐like morphology typical of astrocytes (Figure 1B,E–H) (von Lenhossék 1895; Khakh 2025). Furthermore, this approach consistently achieved sparse and highly reproducible labeling across the different layers of the CA1 region (Figure 1C,D). Using the estimate of ~1500 GFAP‐positive astrocytes per dorsal CA1 subfield in a 50‐μm‐thick adult mouse coronal section (Ogata and Kosaka 2002), we estimate that PALE labels approximately 1%–5% of CA1 astrocytes. When PALE was performed in the same pups at P0 and P1 with plasmids encoding different fluorescent proteins, astrocytes labeled by the distinct electroporation were routinely observed, demonstrating the robustness of this labeling method. Interestingly, only a small number of cells were double‐transfected. (Figure S2A). The expression of the construct already appeared at P5 (Figure S2B), when labeled cells are still in their transitional and migratory phases without the characteristic ramified morphology of fully developed astrocytes (Ge et al. 2012).

To evaluate the cell‐type specificity of hippocampal PALE, we performed fluorescence immunolabeling. All transfected cells expressed the astrocytic marker glial fibrillary acidic protein (GFAP) and glutamine synthetase (GS) within the CA1 subfield (Figure 1E,F), but none of them were positive for the neuronal marker NeuN or the microglial marker Iba1 (Figure 1G,H). Individual astrocytes displayed highly variable morphologies shaped by their local cellular environment. Astrocytes in the dendritic layers exhibited a bushy morphology, whereas those embedded within the pyramidal cell layer extended processes intermingled among pyramidal cell somata. Fibrous astrocytes were located along the corpus callosum.

**FIGURE 2:**
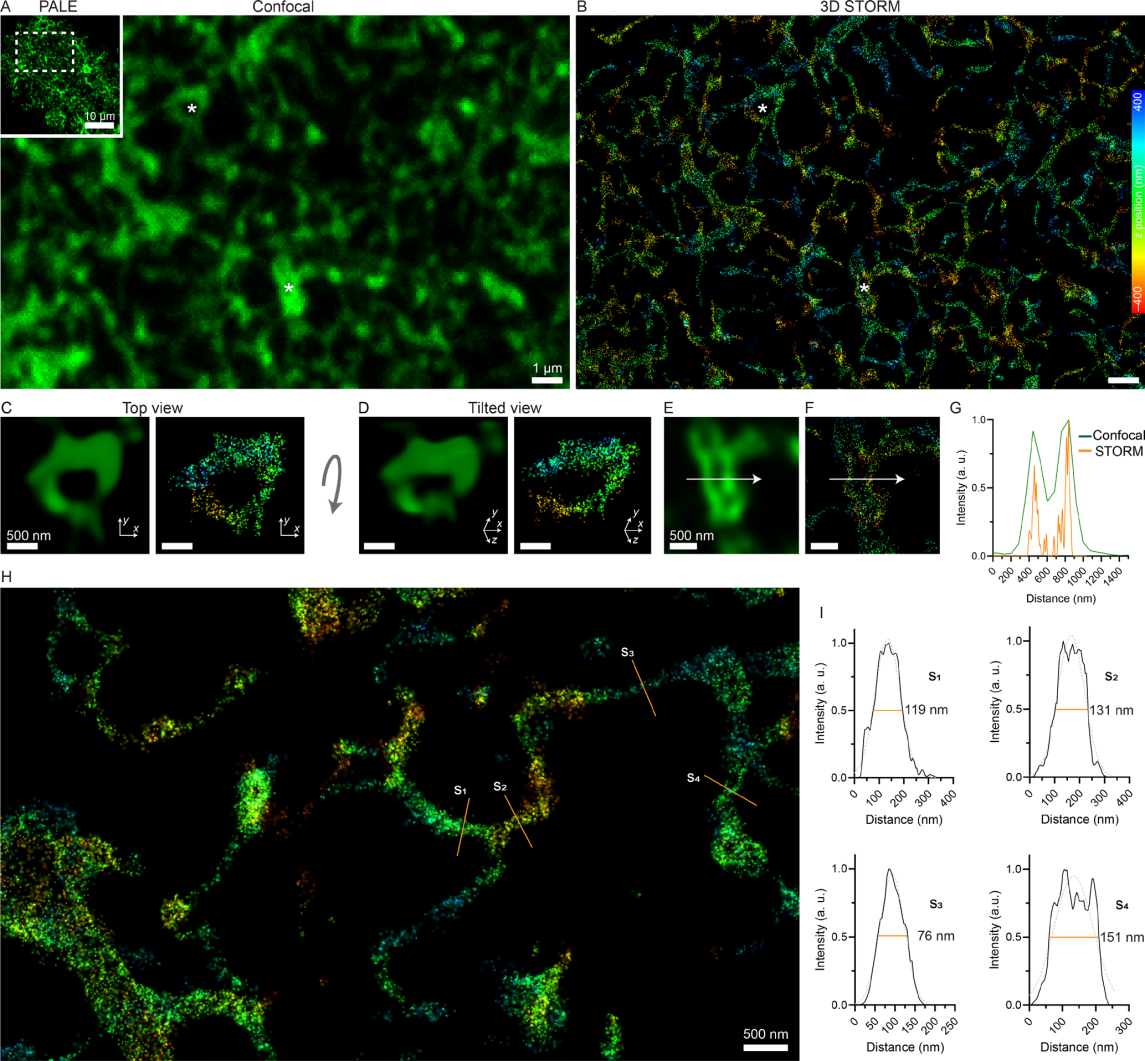
3D‐STORM super‐resolution imaging readily outlines ultrathin astrocytic processes beyond the diffraction limit. (A) High magnification confocal image of a single electroporated astrocyte. Stars denote features that are shown on subsequent figure panels. (B) 3D STORM super‐resolution image of the very same field of view. Color coding is based on the axial depth of localization points relative to the focal plane. Stars denote features that are shown on subsequent figure panels. (C) Enlarged view of a loop‐like structure from panel (A) and (B). The confocal image was deconvolved and 3D reconstructed from 3 planes, corresponding to the range of STORM imaging. (D) Tilted view of the very same structure as in (C). STORM reveals that this loop is only partial and non‐continuous along the *z* direction. (E–G) Intensity measurement along the same structure from corresponding confocal and STORM image pairs (labeled by stars in A and B). (H) STORM imaging of another PALE‐labeled astrocyte, but electroporated with an alternative membrane‐targeted construct, Lck‐GFP. The fine morphological details are similar. (I) Intensity measurements along astrocytic segments (“S”, orange lines in H). Process width is determined from the Full Width at Half Maximum (FWHM) values.

High‐magnification confocal images revealed the general morphology of the astrocytic arbor (Figure 2A). On the other hand, the thinner processes appeared to be blurred and unresolvable even after deconvolution, recalling the notion that the finest astrocytic processes which encompass the majority of the biologically relevant calcium signals (Bindocci et al. 2017; Khakh 2025) fall below the diffraction limit of traditional light microscopy. Therefore, we immunolabeled the membrane‐targeted GFP using primary and secondary antibodies, the latter conjugated to Alexa Fluor 647 (AF647) fluorophores suitable for single‐molecule localization microscopy and switched to STORM super‐resolution microscopy for imaging. Notably, the very same field of view imaged with nanometer precision resolved a sharp outline of even the finest astrocytic processes (Figure 2B). We could also observe striking loop‐like structures that appeared as genuine, continuous loops in confocal z‐stack images (Figure 2C), as described recently using STED microscopy (Arizono et al. 2020, 2021). The superior axial resolution of STORM, however, could reveal that many of these loops were non‐continuous (Aten et al. 2022; Salmon et al. 2023; Baldwin et al. 2024), and only astrocytic segments crossing within the axial dimension created a false impression of continuity (Figure 2D). Intensity measurements showed that the distance at which the intensity of a segment of a thin astrocytic process drops from maximum to background levels is much narrower when using STORM imaging (Figure 2E–G). Thus, the broader intensity profiles seen with confocal microscopy may result in false‐positive assessment of colocalization when imaging closely located non‐astrocytic proteins.

Electroporation of hippocampal astrocytes with an alternative membrane‐targeting construct, Lck‐GFP (Shigetomi et al. 2013) followed by STORM imaging revealed identical ultrastructural anatomical features, demonstrating the generalizability of the approach (Figure 2H). The spongiform astrocytic domains exhibited alternating thin and wide sections on the very same segments. These morphological features are consistent with the “shafts and nodes” or the analogous “constrictions and expansions,” previously described using STED (Arizono et al. 2020) and electron microscopy (Salmon et al. 2023), respectively. Intensity measurements showed that their diameter, quantified by the Full Width at Half Maximum (FWHM) values, was well below the diffraction limit (Figure 2I). In conclusion, the above findings demonstrate that STORM imaging of astrocytes labeled by PALE is a robust method for revealing the astrocytic nanoarchitecture in the intact brain tissue.

### 3.2 Nanoscale Molecular Imaging in Sub‐Diffraction‐Limited Astrocytic Processes

Investigating nanoscale protein distributions in biological structures that are beyond the diffraction limit requires multi‐color STORM imaging. When introducing a second STORM channel, one must ensure that the imaging scheme is free of crosstalk and microscopic aberrations to enable unambiguous data interpretations.

Therefore, we first reconstructed the astrocytic arbor by using a spectrally distinct fluorophore, CF568. When exciting CF568 with its respective high‐power laser line, the abundant PALE‐GFP signal was also weakly excited. Even a small emission elevated the fluorescent background to a level where blinking of the CF568 fluorophores was difficult to discern. Thus, we switched to ChR2‐mCherry as a PALE marker to visualize the cellular context. ChR2‐mCherry was bright enough to validate the success of electroporation without any intensification, and could be easily bleached during STORM imaging, allowing the accurate reconstruction of astrocytes (Figure S3A). To evaluate the degree of channel crosstalk, we imaged the same astrocyte with its non‐specific laser line (647 nm, i.e., the laser line for the other STORM channel). Only a few, non‐structured localization points could be detected, whose number just slightly depended on peak identification stringency (Figure S3B,C). However, this was not the case the other way around (Figure S3D–F). When exciting an AF647‐labeled astrocyte with its non‐specific laser line (561 nm), the astrocytic labeling appeared as a structured low‐intensity fluorescent background, and false localization points were identified with the peak identification settings, producing a channel crosstalk as high as 40%. Simply increasing the stringency for peak identification, however, fully eliminated these artificial localizations (Figure S3E,F), whereas the CF568‐labeled astrocytes could still be reconstructed with adequate detail.

**FIGURE 3:**
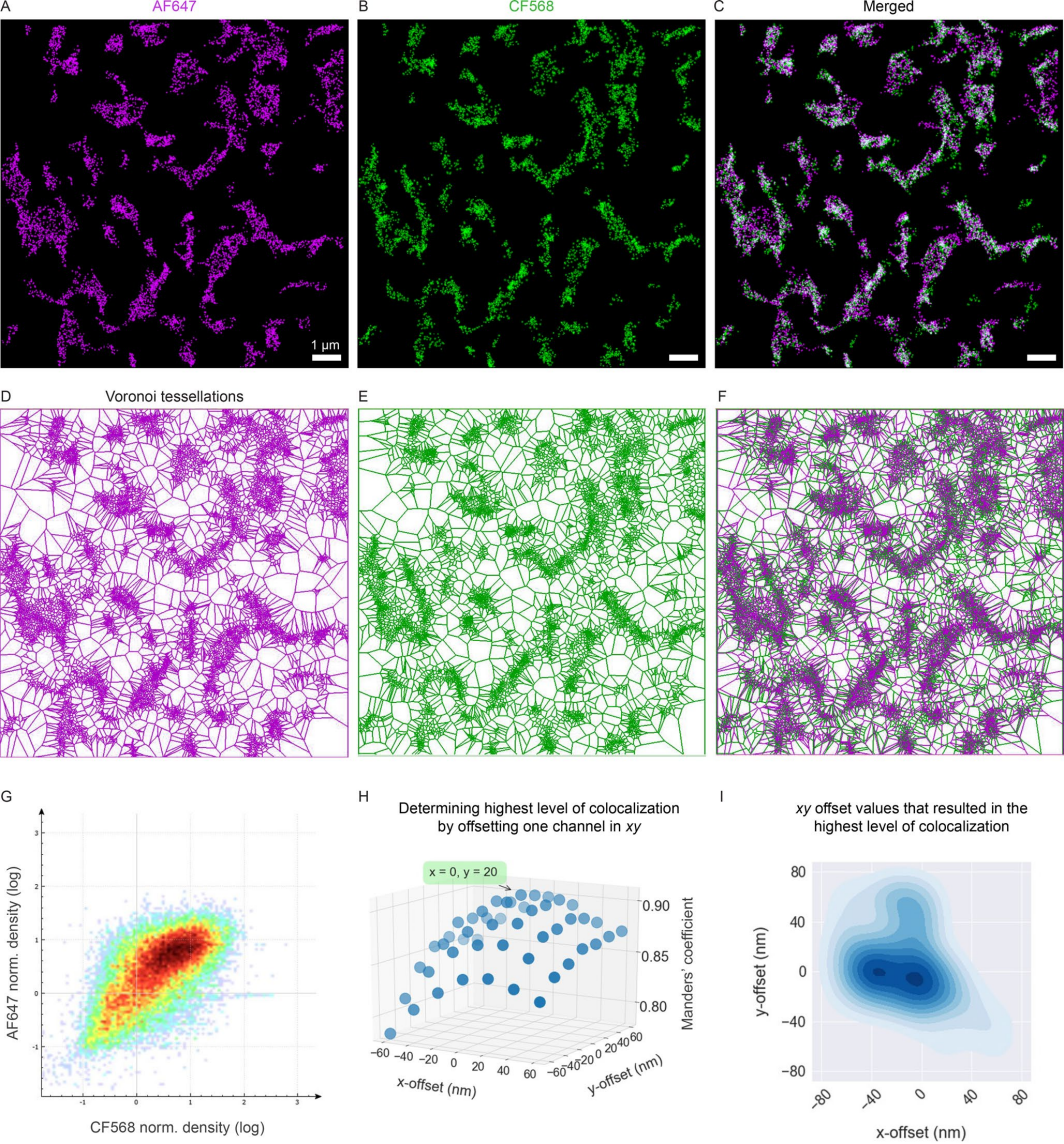
Chromatic aberration‐free imaging of astrocytic processes in dense brain tissue by dual‐color STORM imaging. (A–C) Dual‐color STORM super‐resolution image of an astrocyte labeled with CF568 and AF647 fluorophores, and imaged sequentially, starting with the longer wavelength laser line. (D–F) Voronoi tessellation representation of the STORM localization points for the two images. Tessellation, image creation, calculation of local densities and Manders' values were all performed in Coloc‐Tesseler (Levet et al. 2019). (G) The local molecular densities of the two STORM channels are highly correlated. The Manders' coefficient value that quantifies the level of colocalization can be retrieved from these pair density scatterplots. (H) The AF647 localization points were shifted in *xy* dimensions (−60 to 60 nm, 20 nm increments, independently for *x* and *y* directions) relative to the CF568 ones, and Manders' values were retrieved again. The highest values for this image were detected around 0–20 nm *xy* shifts, and Manders' values started to deteriorate at higher offsets. (I) 2D histogram of the best three Manders' values retrieved from 19 images where one channel was shifted relative to the other using the above parameters. The most frequent values for both the *x* and *y* offsets were around 0 nm. This proves that there is negligible chromatic aberration (and residual drift) between the imaged channels.

Next, we aimed to determine the level of chromatic aberration, that is, the misalignment between channels imaged with lasers of separate colors. Though the imaging of fluorescent TetraSpeck beads attached to coverslips showed negligible chromatic aberration on our setup, this may not necessarily be the case with thicker tissue preparation. Therefore, we labeled the very same astrocytes with two different secondary antibodies, carrying either AF647 or CF568 fluorophores (Figure 3A–C). To quantify the degree of overlap, we used Coloc‐Tesseler that relies on the Voronoi tessellation method to compute the Manders' coefficient value directly from the molecular coordinates and their local densities (Figure 3D–G) (Levet et al. 2015, 2019). Individual dual‐labeled astrocytes showed a very high degree of overlap and shifting one STORM channel relative to the other by 20 nm increments in *xy* dimensions led to a decrease in the Manders' values (Figure 3H). The highest Manders' values appearing at an *xy* shift around (0,0) nm proves that *xy* chromatic aberration, if any, is comparable in magnitude to the localization precision at the imaging depths of a few micrometers in the brain tissue (Figure 3I).

**FIGURE 4:**
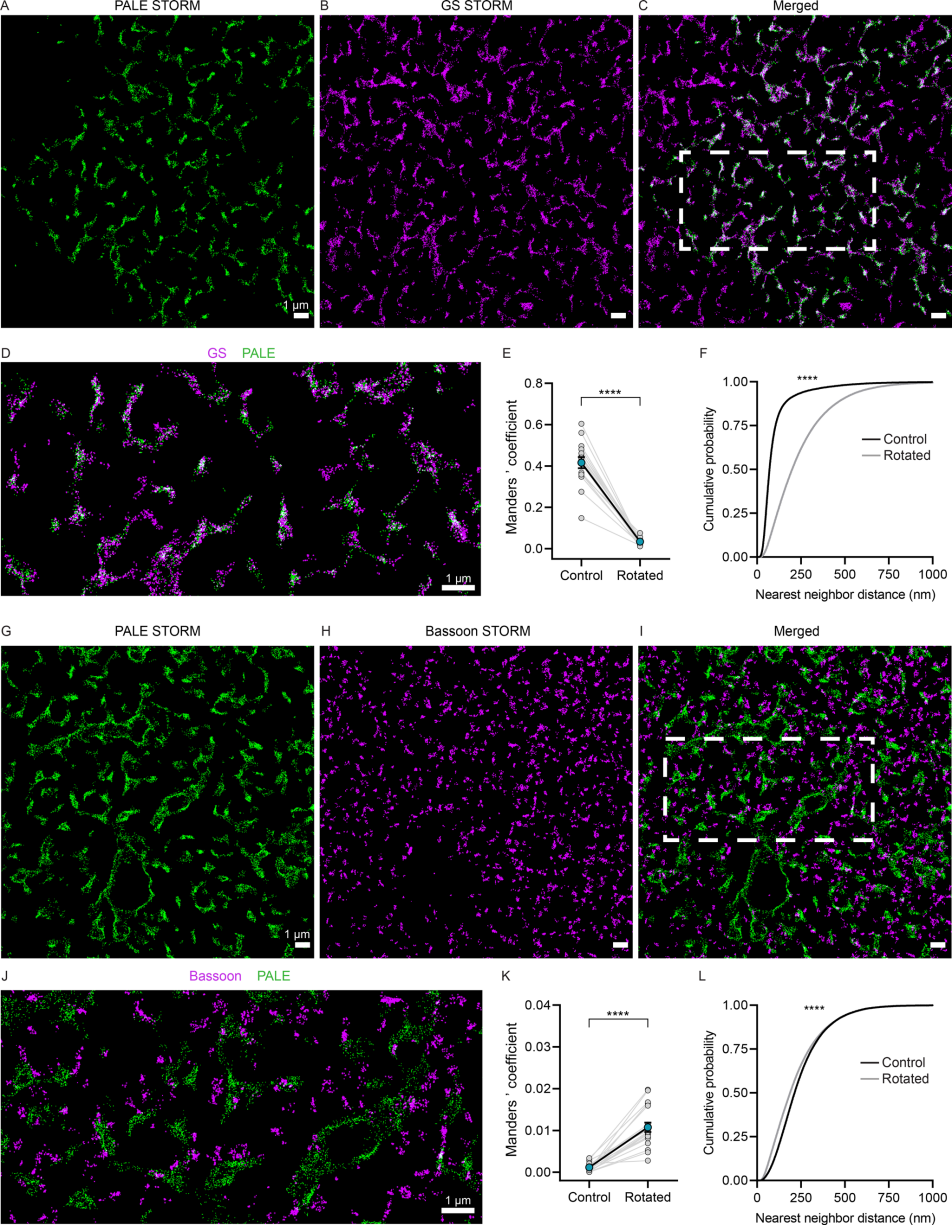
Dual‐color STORM imaging reliably distinguishes between astrocytic and synaptic proteins. (A–C) Dual‐color STORM imaging of the astrocytic nanoanatomy labeled by PALE and the cytosolic glial marker protein glutamine synthetase (GS). (D) Enlarged view of the box in (C) shows a complete overlap between the PALE and GS signal. (E) Manders' coefficient values (obtained from the tessellated super‐resolved images) show the degree of colocalization between the original dual‐color STORM images, but not between their rotated counterparts (*n* = 16 images from 3 animals, paired t‐test, ****p < 0.0001. Mean is shown in cyan with errors as ± SEM). (F) Cumulative distribution function of the nearest neighbor distances between GS and PALE STORM localization points. The distribution for the rotated control image is highly different (*n* = 266,643 localization points from 19 images from 3 animals, two‐sample Kolmogorov–Smirnov test, *d* = 0.28, ****p < 0.0001. Median NND values are 71 nm for the original and 200 nm for the rotated images). (G–I) Dual‐color STORM imaging of the presynaptic active zone protein Bassoon along with the astrocyte. (J) Enlarged view of the box in (I) shows a clear separation between the PALE and Bassoon localization points. (K) Manders' coefficient values indicate zero colocalization between Bassoon and the porated astrocyte. Minimal yet significantly higher Manders' values are observed in the case of rotated controls (*n* = 20 images from 3 animals, Wilcoxon signed‐rank test, ****p < 0.0001. Mean is shown in cyan with errors as ± SEM). (L) NNDs between the Bassoon and PALE localization points show very similar distributions both in the case of the original and rotated image pairs. However, there is a higher occurrence of low NND values on the rotated image (*n* = 170,089 localization points from 23 images from 3 animals, two‐sample Kolmogorov–Smirnov test, *d* = 0.05, ****p < 0.0001. Median NND values are 214 nm for the original and 188 nm for the rotated images).

To determine whether dual‐color STORM imaging provided sufficient spatial resolution to distinguish molecular targets located in adjacent subcellular compartments, we visualized glutamine synthetase (GS), a cytosolic protein specific to astrocytes, and Bassoon, a presynaptic active zone scaffold protein located in neuronal axon terminals (Figure S4A,B). GS localization points completely overlapped with the PALE localization points outlining the astrocytic plasma membrane (Figure 4A–D). We quantified the level of colocalization by calculating the Manders' coefficient values from the Voronoi tessellated images. As a randomization control, the localization points of the target protein were rotated by 90 degrees. This manipulation caused the initially high Manders' values to drop to nearly 0, demonstrating that the observed colocalization values in the original image are not coincidental (Figure 4E). The distance between the GS localization points and their closest PALE localization points (nearest neighbor distance, NND) was also calculated. Comparing the cumulative distribution function of the original images to their rotated counterparts revealed a strong leftward skew, indicating the dominance of small NND values (Figure 4F). Because astrocytes tile the brain by forming non‐overlapping territories with very limited interdigitation under physiological conditions (Bushong et al. 2002), most GS localizations from the territory of a labeled astrocyte belong solely to that individual cell.

STORM imaging of Bassoon (Figure 4G–J) showed a characteristic clustered pattern representing the presynaptic active zones. Bassoon clusters were often adjacent to perisynaptic astrocytic processes but could be well separated without any apparent colocalization. The near‐zero Manders' values compared to their rotated image pairs demonstrated that the nanoscale Bassoon‐immunolabeling signal does not overlap with astrocytic profiles (Figure 4K). Likewise, the distribution curve of NNDs was similar between the original and rotated images, with the rotated counterparts exhibiting lower NND values even more frequently (Figure 4L). The contrasting behavior of the colocalization and the NND values between astrocytic and non‐astrocytic targets upon image rotation was further corroborated by imaging another pair of target proteins, the glial glutamate transporter GLT‐1 and the neuronal microtubule‐associated protein MAP2 (Figure S5). Taken together, these findings confirm that dual‐color STORM imaging has the required resolution and power to reliably discriminate between astrocytic and synaptic targets in brain tissue samples.

### 3.3 CA1 Hippocampal Astrocytes Express the Endocannabinoid‐Degrading Enzyme MAGL

Most proteins do not exhibit complete segregation between distinct cell types. For example, the endocannabinoid‐degrading enzyme monoacylglycerol lipase (MAGL) (Dinh et al. 2002) is present in both neurons and astrocytes. Moreover, presynaptic MAGL primarily controls synaptic endocannabinoid signaling, whereas astrocytic MAGL is predominantly involved in the generation of neuroinflammatory prostaglandins (Viader et al. 2015). Importantly, prior electron microscopic studies uncovered a high abundance of MAGL in presynaptic boutons in the hippocampal CA1 area (Gulyas et al. 2004; Ludányi et al. 2011), but MAGL levels in astrocytes remained below detection threshold in the CA1 subregion, likely due to the limited antibody penetration in tissue preparations prepared for electron microscopy. Therefore, to test the power of the astrocyte‐specific nanoscale molecular imaging approach, we aimed to visualize MAGL independently within neuronal axon terminals and ultrathin astrocytic processes in the CA1 stratum radiatum.

Because astrocytes are highly heterogeneous at the transcriptomic level (Kwon et al. 2025), we first performed RNAscope in situ hybridization to determine whether all CA1 astrocytes express the mRNA for MAGL (*mgll*). High magnification confocal images showed that all astrocytes visualized by *glul* (mRNA for GS) expressed *mgll* in the CA1 region, albeit at lower levels than in the dentate gyrus (Figure S6). The lack of astrocytic labeling in sections from MAGL knockout (KO) mice confirmed the specificity of the *mgll* probe and the RNAscope signal.

**FIGURE 5:**
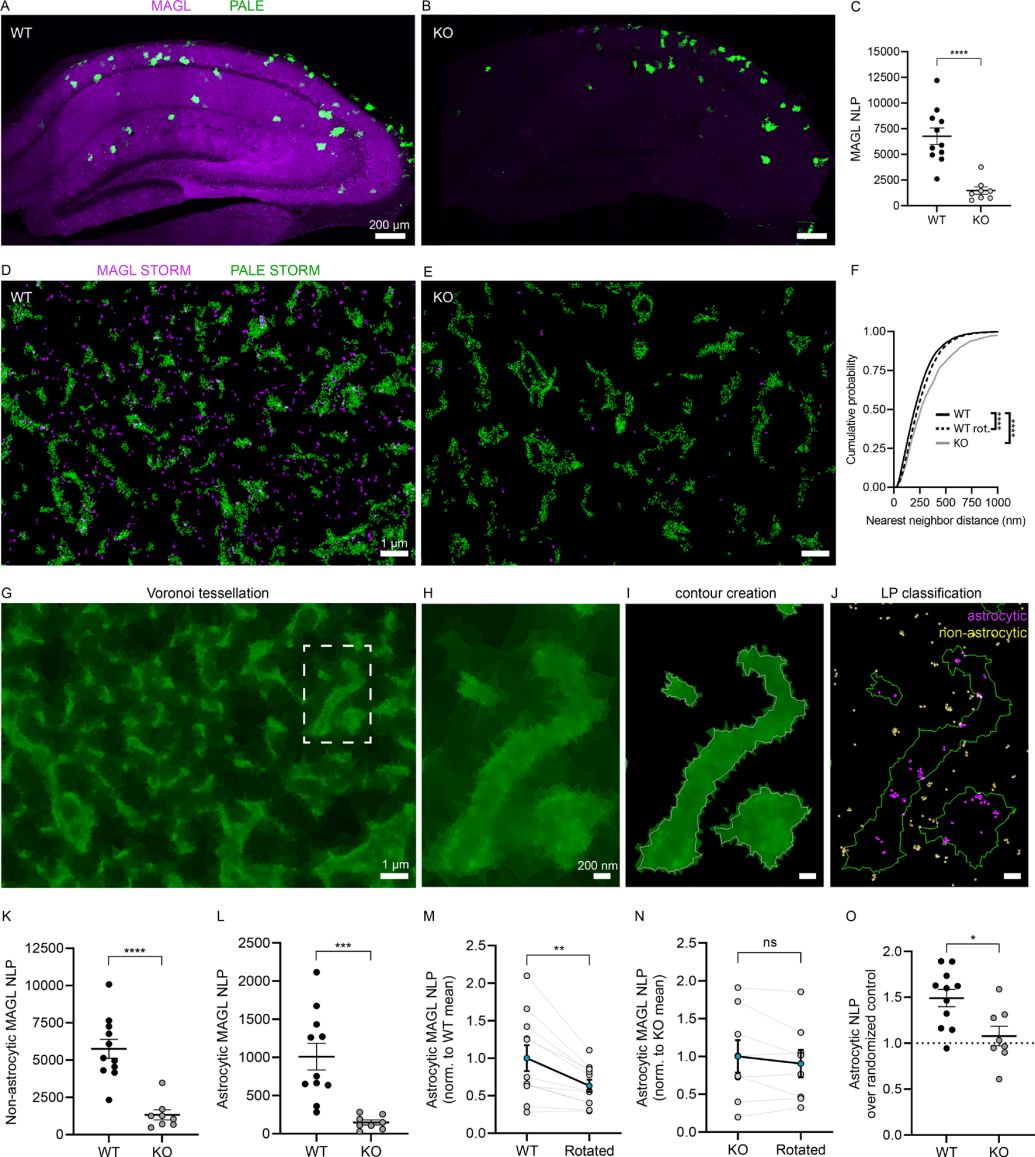
PALE‐based segmentation and STORM‐based single‐molecule detection sensitivity identifies low‐copy‐number MAGL localization points in astrocytes. (A and B) MAGL labeling in MAGL WT and MAGL KO pups with astrocytes labeled by PALE. (C) Quantification of the number of localization points (NLP) in MAGL STORM images (MAGL WT *n* = 11 images from 3 animals, MAGL KO *n* = 8 images from 3 animals, presented as mean ± SEM, unpaired *t*‐test, ****p < 0.0001). (D and E) Dual‐color STORM image of MAGL and the astrocytic arbor obtained from MAGL WT and MAGL KO sections. (F) Distribution of nearest neighbor distances of MAGL and PALE localization points in MAGL WT and KO sections, and in rotated controls of the WT images (WT *n* = 17,994 localization points from 11 images from 3 animals, KO *n* = 2578 localization points from 8 images from 3 animals. WT versus KO, two‐sample Kolmogorov–Smirnov test, *d* = 0.18, ****p < 0.0001; WT versus WT rotated, two‐sample Kolmogorov–Smirnov test, *d* = 0.08, ****p < 0.0001). (G) Voronoi tessellation of PALE STORM from the very same area as in (D). Tessellation and image creation was performed via SR‐Tesseler. (H) Enlarged view of the boxed area in (G). (I) Object creation from Voronoi cells after setting a density factor. The contour of the object will serve as a region of interest (ROI) for subsequent molecule filterings. (J) MAGL STORM localization points are classified as astrocytic and non‐astrocytic ones based on their positioning respective to the astrocytic contours. Localizations were visualized by the Visual Molecular Dynamics (VMD) software. (K and L) Summary graphs of MAGL STORM localization point numbers between MAGL WT and KO animals for non‐astrocytic and astrocytic localizations (*n* = 11 images from WT from 3 animals, and *n* = 8 images from KO from 3 animals, presented as mean ± SEM, unpaired *t*‐test, non‐astrocytic: ****p < 0.0001, astrocytic: ***p < 0.001). (M–O) Summary graphs comparing the astrocytic MAGL NLPs in WT and KO sections between the original and their rotated control image pairs (WT vs. rotated, *n* = 11 images from 3 animals, paired *t*‐test, **p = 0.0034; KO vs. rotated, *n* = 8 images from 3 animals, paired *t*‐test, *p* = 0.2082; fold change over rotated controls, *n* = 11 images from 3 WT animals, and *n* = 8 images from 3 KO animals, unpaired *t*‐test, *p = 0.01. All data are presented as mean ± SEM).

Next, we carried out PALE on MAGL WT and MAGL KO pups. Immunolabeling for MAGL in adult hippocampal sections visualized astrocytes in the molecular layer of the dentate gyrus as described before (Uchigashima et al. 2011). In contrast, immunolabeling in the CA1 subfield resulted in a rather homogeneous pattern (Figure 5A). MAGL‐immunolabeling was absent in hippocampal sections obtained from MAGL KO mice, verifying the specificity of the antibody and the immunolabeling method (Figure 5B). Quantitative STORM imaging in the same sections further verified the specificity of MAGL‐immunolabeling (Figure 5C). Next, we performed dual‐color STORM imaging of MAGL and astrocytes that were labeled by PALE. As a first impression, MAGL was much more abundant in areas devoid of astrocytic processes, consistent with its higher expression in neurons than in astrocytes (Figure 5D). Because it was previously noted that MAGL antibodies can cross‐react with other primary antibodies (Uchigashima et al. 2011), we also took dual‐color STORM images from MAGL KO sections. Only some scattered non‐structured patterns were seen in the knockout sections (Figure 5E), excluding the possibility of antibody cross‐reactivity. We then quantified the NNDs of MAGL and PALE localization points (Figure 5F). The cumulative distribution curve from WT sections did not show a leftward skew as would have been expected for a strong astrocytic enrichment. However, smaller NND values were still more prominent compared to their rotated control images or to images from KO sections, indicating that a small number of MAGL localizations are located within the astrocytes.

To determine what proportion of the MAGL localization points can be attributed specifically to astrocytes, we took advantage of the Voronoi tessellation method (Levet et al. 2015; Lycas et al. 2022). We tessellated the PALE STORM signal (Figure 5G,H), and by setting a density threshold, we created objects with contours delineating the astrocytic arbor (Figure 5I). These contours then served as regions of interest (ROIs) to assign MAGL localizations as either astrocytic or non‐astrocytic ones (Figure 5J). Both non‐astrocytic and astrocytic MAGL localization points were specific as verified in astrocytes obtained from MAGL knockout mice (Figure 5K,L). Notably, astrocytic localization points accounted for only 14% of all MAGL localizations, indicating a much higher abundance of non‐astrocytic, likely mainly presynaptic MAGL compared to astrocytic MAGL (Figure 5K).

To exclude the possibility that non‐astrocytic MAGL localizations were misidentified as astrocytic ones, we compared the astrocytic MAGL density in the original images to their rotated image pairs. Despite the abundant MAGL localizations throughout the neuropil, astrocytic MAGL density was still higher than after image rotation (Figure 5M). This observation demonstrates that MAGL localizations, although sparse, are specifically enriched in astrocytes compared to the neighboring neuropil. Notably, the density of astrocytic localization points in MAGL KO sections was identical to their rotated control images, arguing against antibody cross‐reactivity or the presence of a structured background artifact (Figure 5N,O).

### 3.4 Nanoscale MAGL Distribution in Hippocampal Astrocytes

Cell type‐specific MAGL knockout experiments revealed synaptic and neuroinflammatory roles for MAGL located in neurons and astrocytes, respectively (Viader et al. 2015). Therefore, we aimed to determine whether a distinct nanoscale distribution pattern underlies cell‐type‐specific division of labor.

**FIGURE 6:**
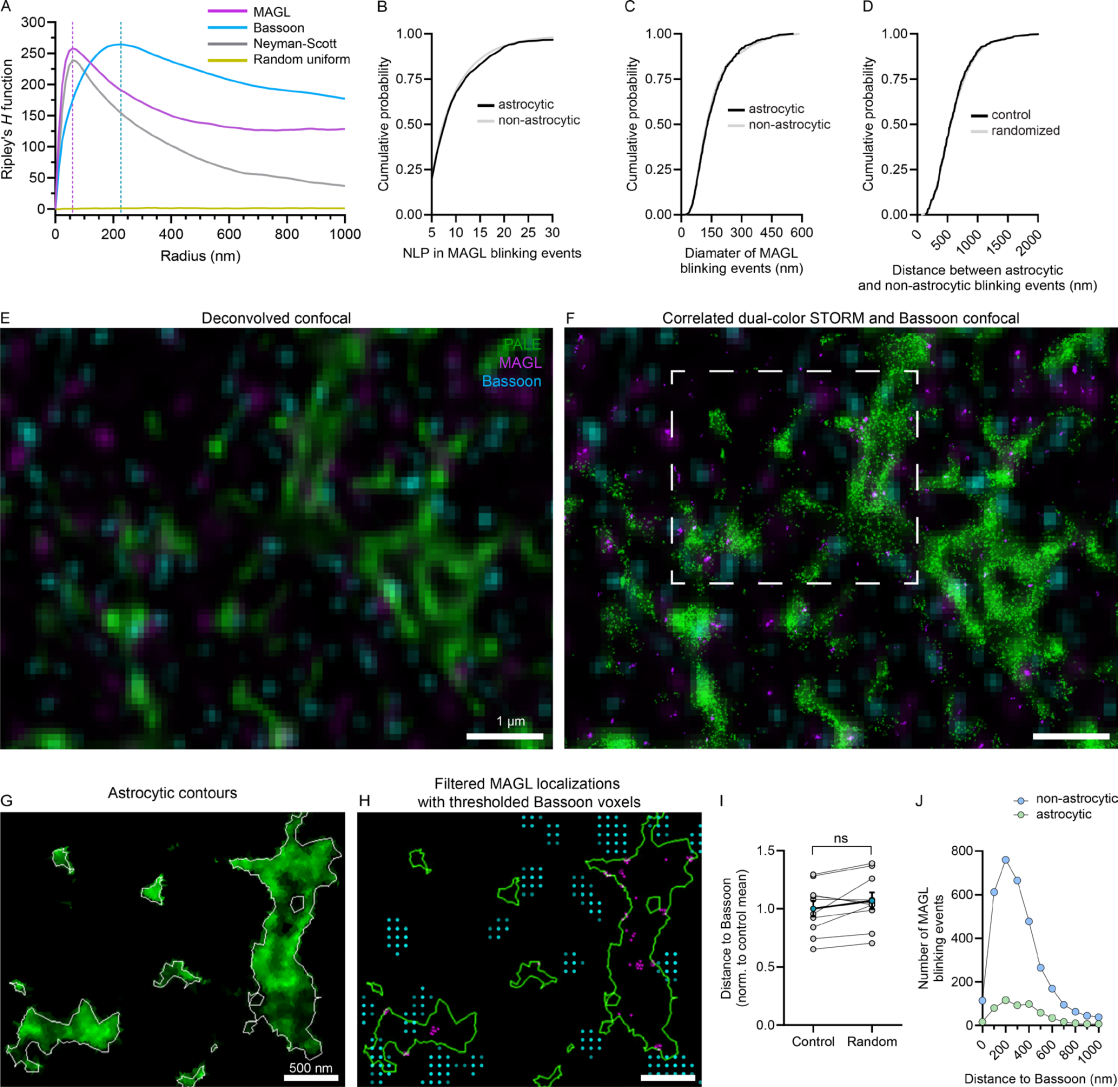
Synapse‐independent nanoscale MAGL localizations are randomly distributed in astrocytic processes. (A) Ripley's *H* function of MAGL and Bassoon. MAGL shows a clustered distribution compared to its randomized control, with a prominent peak around 60 nm, that is much lower than the ~225 nm of the active zone marker protein Bassoon. However, simulated point clusters (Neyman‐Scott process) also have similar spatial characteristics, suggesting that these point clusters arise from multiple blinking during STORM imaging. We will refer to them as “blinking events” in the following. (B) Astrocytic and non‐astrocytic MAGL blinking events are composed of a similar number of localization points (astrocytic *n* = 715 and non‐astrocytic *n* = 4159 blinking events from 10 images from 3 animals, two‐sample Kolmogorov–Smirnov test, *d* = 0.03, *p* = 0.58). Blinking events were identified by the DBSCAN clustering method. (C) Astrocytic and non‐astrocytic MAGL blinking events have similar diameters as determined by the maximal *xy* distance between localization points belonging to the same blinking event (astrocytic *n* = 715 and non‐astrocytic *n* = 4159, two‐sample Kolmogorov–Smirnov test, *d* = 0.04, *p* = 0.38). (D) The nearest inter‐cluster distance between astrocytic and non‐astrocytic MAGL blinking events shows a similar distribution to their randomized controls, where non‐astrocytic blinking events were randomized in the imaging field excluding the astrocytic ROIs (astrocytic *n* = 507 and non‐astrocytic *n* = 4823 blinking events from 11 images from 3 animals, two‐sample Kolmogorov–Smirnov test, *d* = 0.03, *p* = 0.71). (E) High‐resolution deconvolved confocal image of a PALE‐labeled astrocyte, MAGL, and the active zone marker Bassoon. (F) STORM super‐resolution image of the labeled astrocyte and MAGL from the corresponding field of view superimposed onto the Bassoon confocal image. (G) Visualization of astrocytic contours based on the Voronoi tessellation method. (H) Identification of astrocytic MAGL blinking events and synaptic release sites labeled by Bassoon. For clarity, thresholded Bassoon voxels were filtered to 300 nm distance from the focal plane (i.e., the same volume from where the STORM localization points are detected) and are shown as cyan spheres. (I) Comparison of the nearest neighbor distances of MAGL blinking events to Bassoon voxels in control versus randomized datasets (*n* = 10 images from 3 animals, Wilcoxon signed‐rank test, *p* = 0.11. Mean is shown in cyan with errors as ± SEM). (J) Distance distribution of astrocytic and non‐astrocytic MAGL blinking events to Bassoon voxels. The same dataset was analyzed as in (I). Non‐astrocytic localizations were identified as the ones outside of astrocytic contours (*n* = 3377 non‐astrocytic and 546 astrocytic MAGL blinking events).

In general, STORM images of MAGL‐immunolabeling in the hippocampal neuropil depicted a striking clustered pattern. Indeed, when computing the Ripley's *H* function of MAGL STORM images we found that *H*(r) values had a prominent peak around 60 nm, indicating a clustering of the STORM signal at this spatial scale (Figure 6A). Notably, this value is much smaller than the ~225 nm for the active zone marker Bassoon imaged with identical parameters. However, more than one secondary antibody can bind to a primary antibody, secondary antibodies carry ~4–5 fluorophores, and STORM relies on photoswitchable fluorophores that can be detected multiple times. Thus, it is important to emphasize that localization point clusters may arise from the visualization of a single protein (Veatch et al. 2012; Baumgart et al. 2016). Accordingly, randomizing localization point clusters with daughter localizations scattered around the parent localization with a standard deviation matching our localization precision (known as the Neyman‐Scott process) (Siddig et al. 2020) also showed a peak around 60 nm on the Ripley's *H* curve. Importantly, the nanoscale quantification in the neuropil indicates that the majority of MAGL proteins are scattered individually, and the clustering of the MAGL localization points did not arise from true molecular clusters but from multiple blinking events. To avoid any misinterpretation, we will refer to these clustered localization points as “blinking events.” On the other hand, the spatial characteristics of MAGL blinking events may exhibit cell‐type specificity. To address this possibility, we first identified individual blinking events by the DBSCAN algorithm. Despite the 7 times lower overall MAGL densities in astrocytes compared to neurons, neither the number of localization points in individual blinking events (Figure 6B), nor their diameter (Figure 6C) was different compared to the non‐astrocytic MAGL blinking events.

A single rodent astrocyte covers ~100,000 synapses and MAGL plays an essential role in controlling synaptic 2‐AG signaling (Bushong et al. 2002; Pan et al. 2011). Therefore, we next explored if the spatial distribution of the astrocytic MAGL localization points presents any bias towards the putative presynaptic localization points, potentially indicating a shared function in controlling synaptic 2‐AG signaling in the CA1 region. We measured the distance from each astrocytic MAGL blinking event to its nearest non‐astrocytic blinking event (inter‐cluster distance between the centers of mass) and then repeated this step when astrocytic blinking events were randomly redistributed within their respective ROIs. Interestingly, cumulative distribution functions of the inter‐cluster distances were similar between the original and the randomized MAGL blinking event positions, suggesting that there is no preferential targeting of astrocytic MAGL localizations to the multipartite synapse (Figure 6D) (Hodebourg et al. 2025).

To provide independent evidence that the nanoscale distribution of astrocytic MAGL localization points is not associated with synapses, we also performed correlated confocal imaging (Barna et al. 2016; Zöldi and Katona 2023) of the active zone marker protein Bassoon (Figure 6E) and astrocytic MAGL localization points within PALE‐based super‐resolved astrocyte processes (Figure 6F). For each blinking event representing MAGL‐immunolabeling in astrocytes, we measured the distance to the nearest synapse identified from deconvolved images of Bassoon‐immunolabeling of the presynaptic active zone (Figure 6G,H). Notably, when this step was repeated for the randomized MAGL distributions, we found that both the original and randomized MAGL localizations had similar distance distributions to synapses (Figure 6I). These findings demonstrate that, in contrast to the higher density of non‐astrocytic MAGL in the vicinity of Bassoon (Figure 6J), astrocytic MAGL is not enriched around synapses but rather scattered randomly throughout astrocytic processes, suggesting a synapse‐independent functional role.

## 4 Discussion

Due to their complex morphological structures and ultrathin processes, astrocytes are among the most challenging cell types for molecular investigations in the brain. Thus, our understanding of how subcellular compartment‐specific molecular changes mediate functional and pathological processes in astrocytes remains limited compared to neurons. Recent scRNA‐seq studies have greatly contributed to the emerging notion that the molecular repertoire of astrocytes is highly heterogeneous and surprisingly dynamic (Chai et al. 2017; Serrano‐Pozo et al. 2024; Hennes et al. 2025; Kwon et al. 2025; O'Dea and Hasel 2025; Schroeder et al. 2025). However, these studies lack subcellular compartment‐specific resolution, and low‐copy transcripts may also remain undetected, particularly those that are locally translated in distal processes (Sakers et al. 2017). Moreover, mRNA levels often fail to correlate with actual protein abundance (Soto et al. 2023).

Historically, electron microscopy (EM) has been the gold standard for subcellular localization; however, EM often fails to detect low‐density molecules due to labeling constraints. Furthermore, the harsh dehydration and fixation required for EM elicits anisotropic shrinkage in the extracellular space thereby introducing structural artifacts, such as altered synaptic coverage, leading to the misinterpretation of molecular distances (Korogod et al. 2015). Super‐resolution microscopic techniques address these limitations by offering high sensitivity without the need for EM‐specific sample preparation. While STED and expansion microscopy (ExM) approaches have successfully resolved astrocytic architecture (Arizono et al. 2020; Herde et al. 2020; Arizono and Nägerl 2022), SMLM techniques such as STORM imaging offer a unique advantage for measuring nanoscale molecular heterogeneities. Indeed, by exploiting this potential, prior SMLM imaging studies in astrocytes could successfully visualize highly abundant proteins that are exclusively expressed by astrocytes (Heller et al. 2017, 2020; Henneberger et al. 2020; Aleksejenko and Heller 2021). However, most essential signaling proteins occur in several cell types, not only in astrocytes, and are restricted to specific nanodomains within ultrathin astrocytic processes, thereby necessitating the visualization of the complete outline of the astrocytic plasma membrane in intact brain circuits. While technically less demanding, SMLM imaging in cell culture systems has also limitations, because astrocytes adopt inflammatory (“reactive”) phenotypes and exhibit molecular signatures that strongly differ in the healthy brain (Cahoy et al. 2008; Escartin et al. 2021). To circumvent these obstacles, using dual‐color STORM imaging, we were able to reconstruct the morphology of individual hippocampal astrocytes and to perform distance measurements within their diffraction‐limited fine processes—both with nanoscale precision. We also introduced solutions to measure, analyze, and visualize nanoscale molecular distributions in specific astrocytic microdomains together with a battery of essential control experiments that can be extended to any molecular target by reproducing the workflow.

These approaches enabled us to readily distinguish between the abundant neuronal and the relatively scarce astrocytic pools of the endocannabinoid‐degrading enzyme MAGL. MAGL is a unique serine hydrolase that controls synaptic 2‐AG signaling and metabolizes 2‐arachidonoyl‐glycerol to arachidonic acid that serves as the substrate for neuroinflammatory prostaglandin production (Nomura et al. 2011; Chen 2023). The latter function is mediated by the astrocytic MAGL pool (Viader et al. 2015; Grabner et al. 2016). Interestingly, while the high abundance of MAGL in axon terminals has already been revealed by electron microscopy in the CA1 region (Gulyas et al. 2004; Ludányi et al. 2011), astrocytic MAGL distribution has not yet been investigated in this circuit. Thus, we also implemented a sparse labeling approach specifically for astrocytes in the CA1 subregion of the hippocampus. Surprisingly, we found that astrocytic MAGL localizations were not preferentially positioned near synaptic release sites but were distributed randomly throughout the astrocytic processes. This finding implies that the astrocytic MAGL pool plays a limited role in controlling retrograde 2‐AG signaling in the CA1 region. Instead, the presynaptic MAGL pool is more likely to have a major regulatory function in synaptic endocannabinoid signaling. Importantly, this scenario is consistent with a new study reporting that synaptic 2‐AG signals primarily to neurons, whereas astrocytes are engaged by another endocannabinoid, anandamide (Noriega‐Prieto et al. 2026).

Which additional rules might dictate the subcellular targeting of astrocytic MAGL? Circuit‐wide large surges in 2‐AG concentrations accompany pathological processes such as epileptic seizures in the CA1 region (Farrell et al. 2021). Thus, it is conceivable to hypothesize that the random distribution of MAGL throughout astrocytic processes may reflect that its substrate, 2‐AG, reaches the enzyme in a non‐synaptic volumetric manner. Moreover, the broad distribution may also be consistent with the role of the astrocytic MAGL pool in the production of prostaglandins, particularly PGE_2_, which has widespread vasoconstricting effects on parenchymal arterioles via EP1 receptor activation (Farrell et al. 2021). The astrocyte‐specific nanoscale molecular imaging approach presented here will open the way for future studies addressing whether astrocytic MAGL proteins are organized into signalosome nanodomains with the downstream enzymes of the prostaglandin pathway, such as the cyclooxygenases (COXs).

Besides neuronal and vascular coupling, there is also important crosstalk between astrocytes and microglia, especially during inflammation (Linnerbauer et al. 2020; Diaz‐Castro et al. 2021). Of note, astrocyte‐specific MAGL deletion alters the mRNA expression profile of inflammation‐related genes to a greater extent in microglia than in astrocytes themselves (Zhu, Zhang, Hashem, et al. 2023). Therefore, the fine‐tuning of prostaglandin levels may involve complex, multicellular pathways mediated by the shuttling of lipid metabolites. Investigating whether specialized contact sites underlie intercellular lipid signaling (Cheung et al. 2022; de Ceglia et al. 2023) will also be possible by applying the methodological approaches introduced here. Finally, while we used an immunolabeling method in the present study, an exciting future direction can be the application of fluorescent small molecule inhibitors such as activity‐based fluorescent MAGL probes (Prokop et al. 2021; van der Vliet et al. 2025). These tools could provide further insights into cell‐type‐specific and nanodomain‐restricted profiles of enzymatic activity under various physiological and pathological conditions.

## Author Contributions

M.Z. carried out postnatal electroporation, immunolabeling experiments, designed and performed confocal and STORM imaging, analyzed the data, prepared the figures and wrote the manuscript. I.K. conceived and supervised the project and wrote the manuscript.

## Funding

This study was supported by the National Institutes of Health (P30DA056410) and by the NKFIH EXCELLENCE program (151377). I.K. holds the Naus Family Chair in Addiction Sciences in the Department of Psychological and Brain Sciences at Indiana University Bloomington.

## Conflicts of Interest

The authors declare no conflicts of interest.

## Supporting information


**Figure S1:** Postnatal electroporation labels diverse cell types. By changing the position of the electrodes cortical astrocytes (A), astrocytes and neural stem cells along the lateral ventricles (B), and olfactory bulb interneurons (C) can also be sparsely labeled by postnatal electroporation.

**Figure S2:** Applications of PALE for sequential temporal labeling and targeting of astrocyte precursors. (A) PALE was performed on two consecutive days using plasmid constructs expressing different fluorescent proteins (P0: GFAP‐EGFP; P1: GFAP‐ChR2‐mCherry). Note that most of the labeled cells were positive only for one of the two constructs. The boxed area is enlarged in the upper right corner, highlighting an astrocyte that was double transfected. (B) PALE labeling appears as early as P5. Note that labeled cells are still in their transitional and migratory phases; their characteristic ramified astrocytic morphology and functional maturation only begin to elaborate after P7.

**Figure S3:** Adjusting peak identification thresholds carefully enables crosstalk‐free multicolor STORM imaging. (A) STORM super‐resolution image of an astrocyte labeled with the CF568 fluorophore and excited with its respective (561 nm) laser line. (B) Exciting the very same astrocyte with the other, non‐specific laser line (647 nm) only results in a few, non‐structured localization points, whose number only minimally decrease with more stringer identification parameters (“th” refers to threshold for peak identification). (C) Quantification of crosstalk at different threshold parameters for the non‐specific laser line. Crosstalk was determined from localization point number ratios obtained from imaging the very same astrocyte with its specific and non‐specific laser line. Background localization point numbers (obtained from areas where no labeled astrocyte was located) were subtracted from the actual localization point numbers. Data are from 12 images, and are presented as median with IQR. (D–F) Same as (A–C), just labeling the astrocyte with AF647 fluorophore, and hence the naming of specific (647 nm) and non‐specific (561 nm) laser lines are changed accordingly. (D) AF647‐labeled astrocytes show superior image quality compared to the CF568‐labeled ones. (E) At lower threshold settings, there is a substantial crosstalk when exciting the AF647‐labeled astrocyte with its non‐specific laser line. This crosstalk can be greatly reduced by elevating the thresholding stringency. (F) Summary graph of the crosstalk at different thresholding parameters for the AF647‐labeled astrocyte excited with the 561 nm laser line. Data are from 9 images, and are presented as median with IQR.

**Figure S4:** Confocal and STORM imaging of astrocytes labeled by PALE. (A) Confocal image of an astrocyte labeled by PALE and the astrocyte marker glutamine synthetase (GS). The STORM super‐resolution image corresponding to the boxed area is shown on the right. (B) Same as in (A), showing the presynaptic active zone marker Bassoon alongside the PALE labeling.

**Figure S5:** Dual‐color STORM imaging reliably distinguishes between astrocytic and non‐astrocytic targets. (A–C) Dual‐color STORM imaging of the astrocytic nanoanatomy labeled by PALE and the plasma membrane‐bound glial marker protein glutamate transporter (GLT‐1). (D) Enlarged view of the box in (C) shows a high overlap between the PALE and GLT‐1 signal. (E) Manders' coefficient values (obtained from the tessellated super‐resolved images) show the degree of colocalization between the original dual‐color STORM images, but not between their rotated counterparts (*n* = 15 images from 1 animal, paired *t*‐test, ****p < 0.0001. Mean is shown in cyan with errors as ± SEM). (F) Cumulative distribution function of the nearest neighbor distances between GLT‐1 and PALE STORM localization points. The distribution for the rotated control image is highly different (*n* = 75,837 localization points from 15 images from 1 animal, two‐sample Kolmogorov–Smirnov test, *d* = 0.22, ****p < 0.0001. Median NND values are 115 nm for the original and 317 nm for the rotated images). (G–J) Dual‐color STORM imaging of the astrocytic nanoanatomy and the neuronal microtubule‐associated protein 2 (MAP2). (K) MAP2 does not co‐localize with astrocytes, and the rotated controls have even slightly higher colocalization values (*n* = 16 images from 1 animal, Wilcoxon signed‐rank test, ***p < 0.0002. Mean is shown in cyan with errors as ± SEM). (L) Rotated control images have slightly lower NND values compared to the original ones (*n* = 83,180 localization points from 18 images from 1 animal, two‐sample Kolmogorov–Smirnov test, *d* = 0.03, ****p < 0.0001. Median NND values are 328 nm for the original and 300 nm for the rotated images).

**Figure S6:** All CA1 astrocytes express the *mgll* mRNA. (A and B) RNAscope in situ hybridization against *mgll* (MAGL) and *glul* (GS) in MAGL WT and KO mice. Note that the majority of *mgll* RNAscope signal disappears in KO sections, showing the specificity of the probe. (C and D) Higher magnification images from the CA1 demonstrate that all CA1 astrocytes express *mgll*, although the signal is much less intensive than in the dentate gyrus. The abundant neuropil *mgll* expression (either axonal or astrocytic local mRNA translation) also disappears in the KO. (E and F) Higher magnification images from the dentate gyrus show abundant *mgll* signal along the somata and main branches of astrocytes labeled by *glul* in MAGL WT sections.

## Data Availability

The data that support the findings of this study are available from the corresponding author upon reasonable request.
