# Supplementary figures and images for: A Super‐Resolution Approach for Astrocyte‐Specific Molecular Imaging Reveals the Nanoscale Distribution of Monoacylglycerol Lipase, the Metabolic Node Between Endocannabinoid and Prostaglandin Signaling
Source: Glia. 2026 Jul 3;74(9):e70186. doi: 10.1002/glia.70186 (PMC13330557; doi:10.1002/glia.70186)

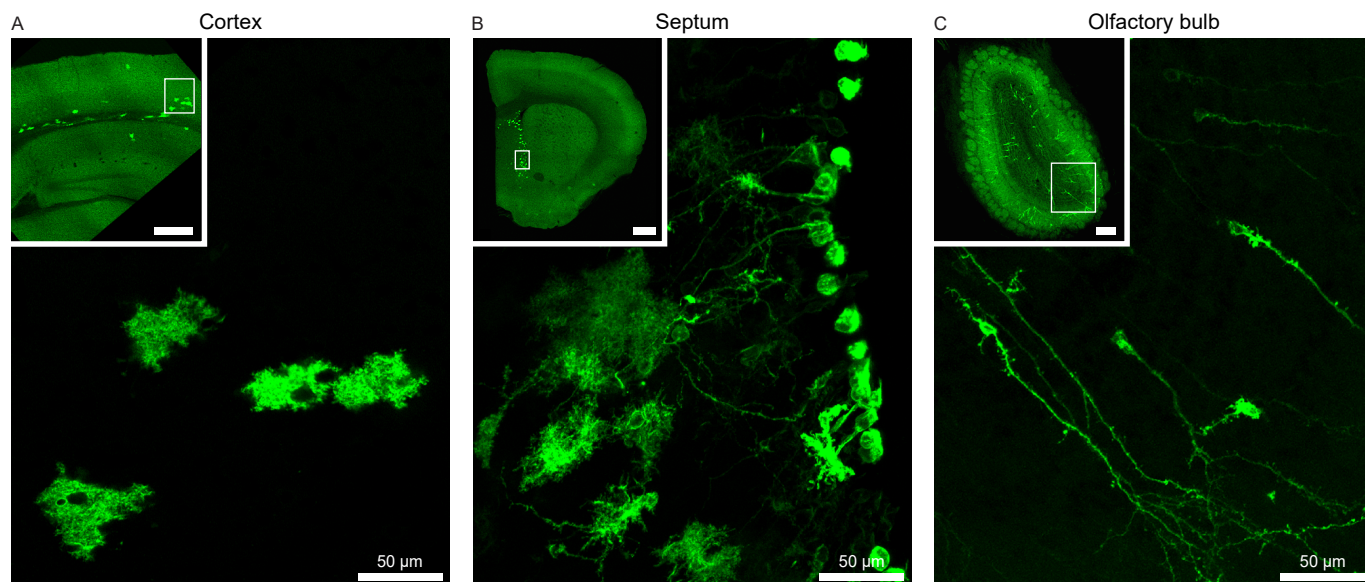

**Figure S1**  
**Zöldi and Katona, 2026**

**Postnatal electroporation labels diverse cell types.**

Supplement: Supplementary file 1 — Figure S1: Postnatal electroporation labels diverse cell types. By changing the position of the electrodes cortical astrocytes (A), astrocytes and neural stem cells along the lateral ventricles (B), and olfactory bulb interneurons (C) can also be sparsely labeled by postnatal electroporation. [file GLIA-74-0-s005.pdf]

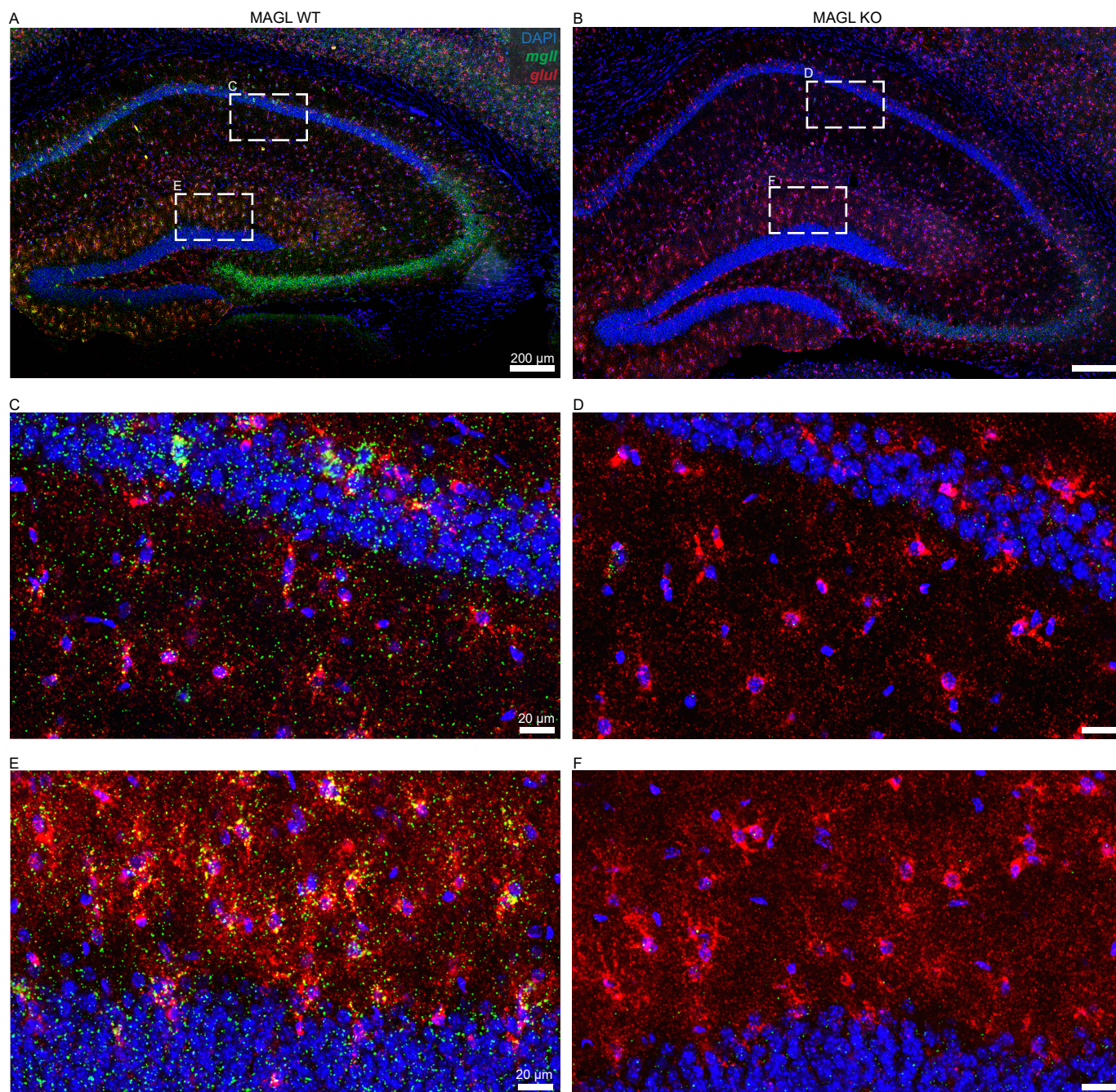

**Figure S6**  
Zöldi and Katona, 2026

All CA1 astrocytes express the *mgl1* mRNA.

Supplement: Supplementary file 6 — Figure S6: All CA1 astrocytes express the mgll mRNA. (A and B) RNAscope in situ hybridization against mgll (MAGL) and glul (GS) in MAGL WT and KO mice. Note that the majority of mgll RNAscope signal disappears in KO sections, showing the specificity of the probe. (C and D) Higher magnification images from the CA1 demonstrate that all CA1 astrocytes express mgll, although the signal is much less intensive than in the dentate gyrus. The abundant neuropil mgll expression (either axonal or astrocytic local mRNA translation) also disappears in the KO. (E and F) Higher magnification images from the dentate gyrus show abundant mgll signal along the somata and main branches of astrocytes labeled by glul in MAGL WT sections. [file GLIA-74-0-s002.pdf]
