# Supplementary material for: A Super‐Resolution Approach for Astrocyte‐Specific Molecular Imaging Reveals the Nanoscale Distribution of Monoacylglycerol Lipase, the Metabolic Node Between Endocannabinoid and Prostaglandin Signaling
Source: Glia. 2026 Jul 3;74(9):e70186. doi: 10.1002/glia.70186 (PMC13330557; doi:10.1002/glia.70186)
Supplement: Supplementary file 2 — Figure S2: Applications of PALE for sequential temporal labeling and targeting of astrocyte precursors. (A) PALE was performed on two consecutive days using plasmid constructs expressing different fluorescent proteins (P0: GFAP‐EGFP; P1: GFAP‐ChR2‐mCherry). Note that most of the labeled cells were positive only for one of the two constructs. The boxed area is enlarged in the upper right corner, highlighting an astrocyte that was double transfected. (B) PALE labeling appears as early as P5. Note that labeled cells are still in their transitional and migratory phases; their characteristic ramified astrocytic morphology and functional maturation only begin to elaborate after P7. [file GLIA-74-0-s006.pdf]

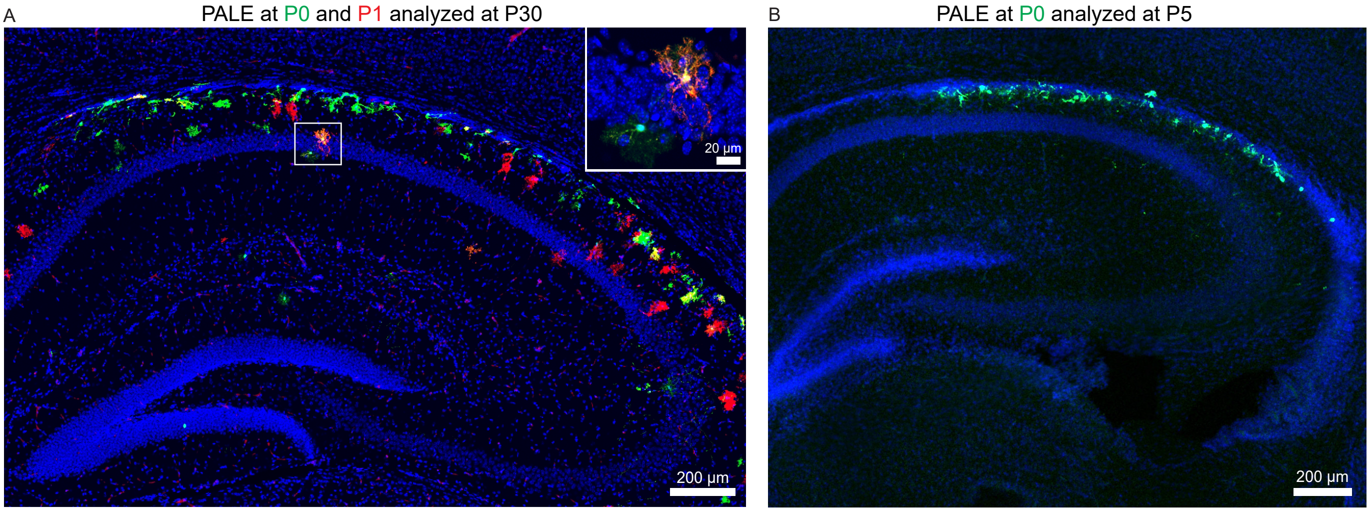

Figure S2
Zöldi and Katona, 2026

Applications of PALE for sequential temporal labeling and targeting of astrocyte precursors.
