# Supplementary material for: A Super‐Resolution Approach for Astrocyte‐Specific Molecular Imaging Reveals the Nanoscale Distribution of Monoacylglycerol Lipase, the Metabolic Node Between Endocannabinoid and Prostaglandin Signaling
Source: Glia. 2026 Jul 3;74(9):e70186. doi: 10.1002/glia.70186 (PMC13330557; doi:10.1002/glia.70186)
Supplement: Supplementary file 3 — Figure S3: Adjusting peak identification thresholds carefully enables crosstalk‐free multicolor STORM imaging. (A) STORM super‐resolution image of an astrocyte labeled with the CF568 fluorophore and excited with its respective (561 nm) laser line. (B) Exciting the very same astrocyte with the other, non‐specific laser line (647 nm) only results in a few, non‐structured localization points, whose number only minimally decrease with more stringer identification parameters (“th” refers to threshold for peak identification). (C) Quantification of crosstalk at different threshold parameters for the non‐specific laser line. Crosstalk was determined from localization point number ratios obtained from imaging the very same astrocyte with its specific and non‐specific laser line. Background localization point numbers (obtained from areas where no labeled astrocyte was located) were subtracted from the actual localization point numbers. Data are from 12 images, and are presented as median with IQR. (D–F) Same as (A–C), just labeling the astrocyte with AF647 fluorophore, and hence the naming of specific (647 nm) and non‐specific (561 nm) laser lines are changed accordingly. (D) AF647‐labeled astrocytes show superior image quality compared to the CF568‐labeled ones. (E) At lower threshold settings, there is a substantial crosstalk when exciting the AF647‐labeled astrocyte with its non‐specific laser line. This crosstalk can be greatly reduced by elevating the thresholding stringency. (F) Summary graph of the crosstalk at different thresholding parameters for the AF647‐labeled astrocyte excited with the 561 nm laser line. Data are from 9 images, and are presented as median with IQR. [file GLIA-74-0-s003.pdf]

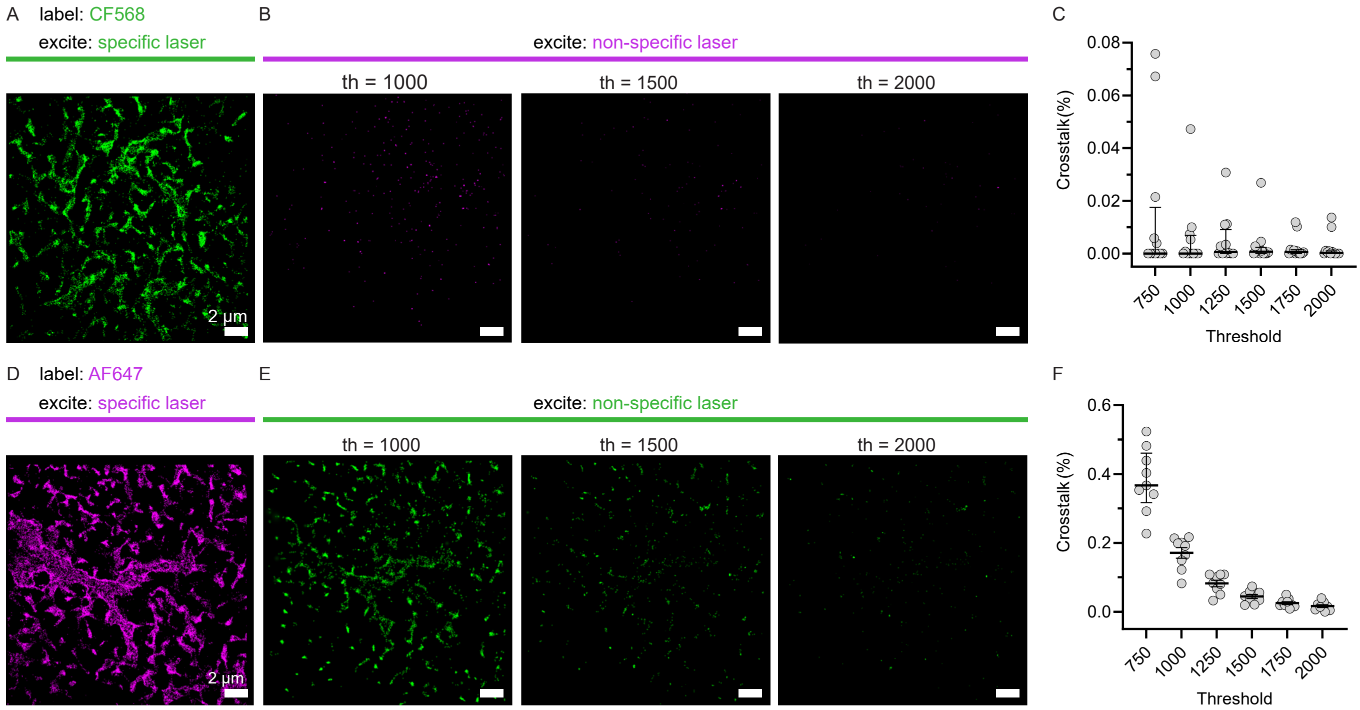

Figure S3
Zöldi and Katona, 2026

Adjusting peak identification thresholds carefully enables crostalk-free multicolor STORM imaging.
