# Supplementary material for: A Super‐Resolution Approach for Astrocyte‐Specific Molecular Imaging Reveals the Nanoscale Distribution of Monoacylglycerol Lipase, the Metabolic Node Between Endocannabinoid and Prostaglandin Signaling
Source: Glia. 2026 Jul 3;74(9):e70186. doi: 10.1002/glia.70186 (PMC13330557; doi:10.1002/glia.70186)
Supplement: Supplementary file 4 — Figure S4: Confocal and STORM imaging of astrocytes labeled by PALE. (A) Confocal image of an astrocyte labeled by PALE and the astrocyte marker glutamine synthetase (GS). The STORM super‐resolution image corresponding to the boxed area is shown on the right. (B) Same as in (A), showing the presynaptic active zone marker Bassoon alongside the PALE labeling. [file GLIA-74-0-s001.pdf]

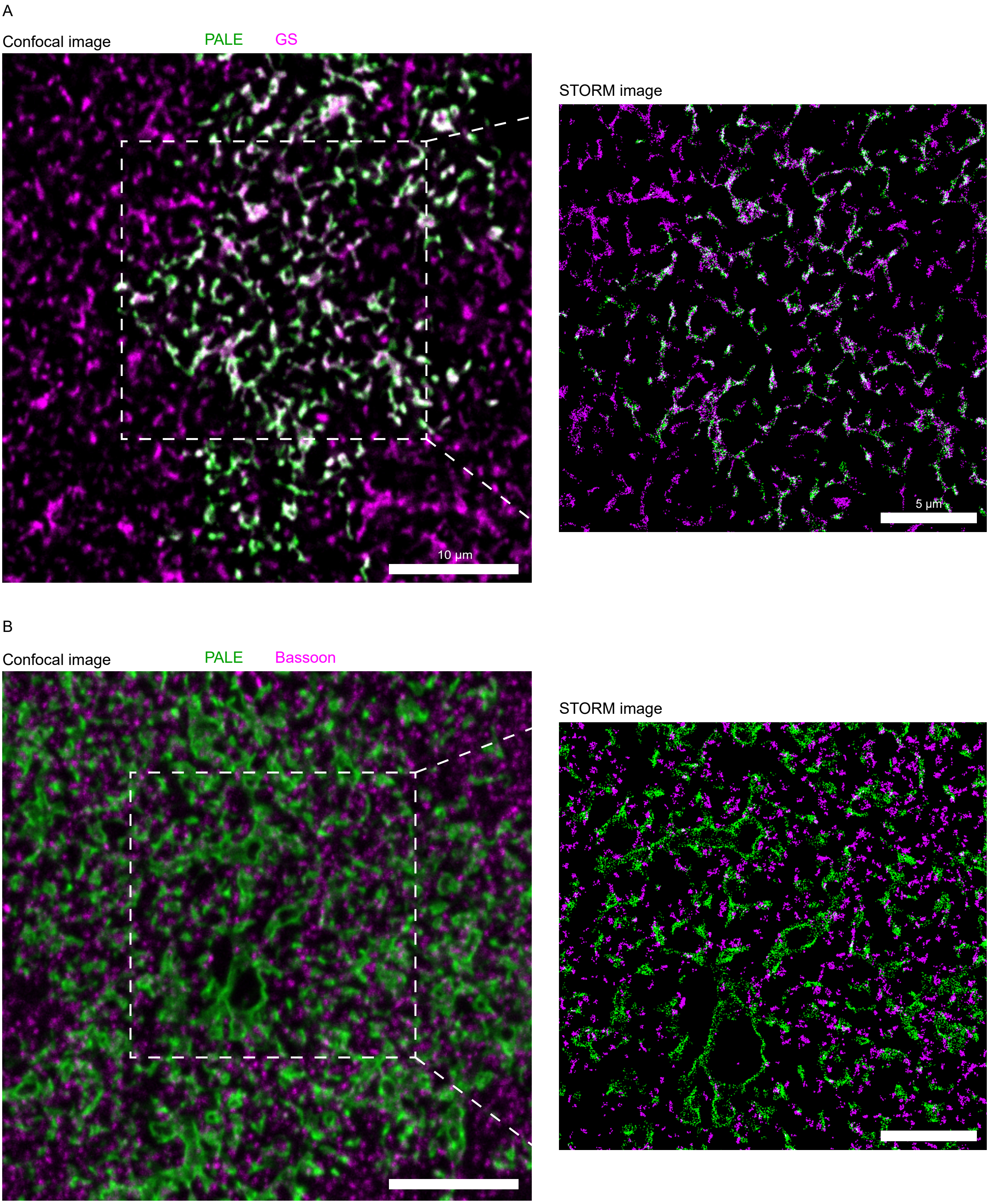

Figure S4
Zöldi and Katona, 2026

Confocal and STORM imaging of astrocytes labeled by PALE.
