# Supplementary material for: A Super‐Resolution Approach for Astrocyte‐Specific Molecular Imaging Reveals the Nanoscale Distribution of Monoacylglycerol Lipase, the Metabolic Node Between Endocannabinoid and Prostaglandin Signaling
Source: Glia. 2026 Jul 3;74(9):e70186. doi: 10.1002/glia.70186 (PMC13330557; doi:10.1002/glia.70186)
Supplement: Supplementary file 5 — Figure S5: Dual‐color STORM imaging reliably distinguishes between astrocytic and non‐astrocytic targets. (A–C) Dual‐color STORM imaging of the astrocytic nanoanatomy labeled by PALE and the plasma membrane‐bound glial marker protein glutamate transporter (GLT‐1). (D) Enlarged view of the box in (C) shows a high overlap between the PALE and GLT‐1 signal. (E) Manders' coefficient values (obtained from the tessellated super‐resolved images) show the degree of colocalization between the original dual‐color STORM images, but not between their rotated counterparts (n = 15 images from 1 animal, paired t‐test, ****p < 0.0001. Mean is shown in cyan with errors as ± SEM). (F) Cumulative distribution function of the nearest neighbor distances between GLT‐1 and PALE STORM localization points. The distribution for the rotated control image is highly different (n = 75,837 localization points from 15 images from 1 animal, two‐sample Kolmogorov–Smirnov test, d = 0.22, ****p < 0.0001. Median NND values are 115 nm for the original and 317 nm for the rotated images). (G–J) Dual‐color STORM imaging of the astrocytic nanoanatomy and the neuronal microtubule‐associated protein 2 (MAP2). (K) MAP2 does not co‐localize with astrocytes, and the rotated controls have even slightly higher colocalization values (n = 16 images from 1 animal, Wilcoxon signed‐rank test, ***p < 0.0002. Mean is shown in cyan with errors as ± SEM). (L) Rotated control images have slightly lower NND values compared to the original ones (n = 83,180 localization points from 18 images from 1 animal, two‐sample Kolmogorov–Smirnov test, d = 0.03, ****p < 0.0001. Median NND values are 328 nm for the original and 300 nm for the rotated images). [file GLIA-74-0-s004.pdf]

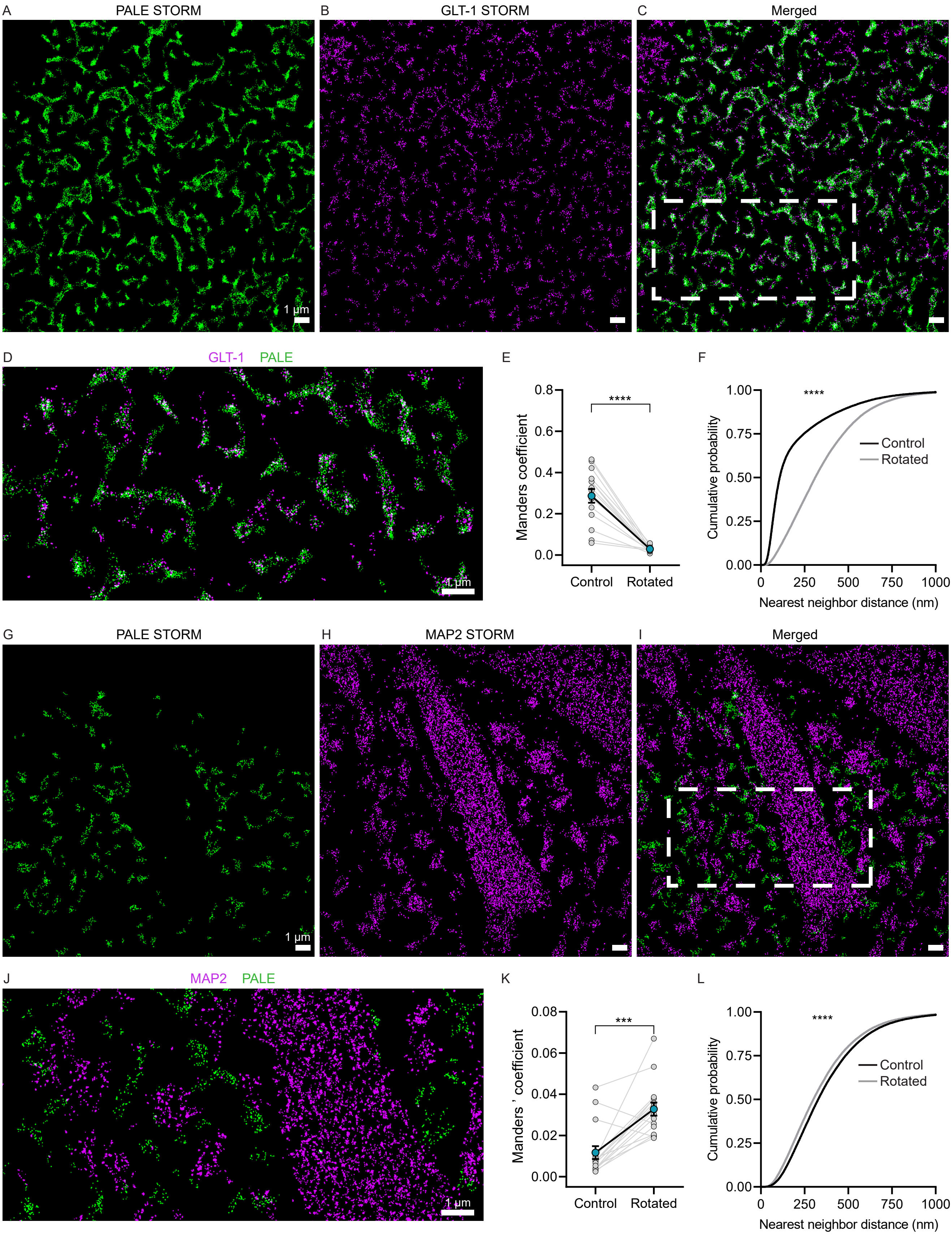

Figure S5
Zöldi and Katona, 2026

Dual-color STORM imaging reliably distinguishes between astrocytic and non-astrocytic targets.
